# Advancing Nitrate‐to‐Ammonia Electrocatalysis: Strategies in Catalyst Design, Electrolyte Engineering, and Performance Evaluation

**DOI:** 10.1002/advs.202508614

**Published:** 2025-07-23

**Authors:** Juan Bai, Ziyou Dong, Xudong Jiang, Qianqin Zhou, Jiahao Zhao, Jun Mei, Ziqing Tan, Ting Liao, Ziqi Sun

**Affiliations:** ^1^ School of Chemistry and Physics Queensland University of Technology 2 George Street Brisbane QLD 4000 Australia; ^2^ Centre for Materials Science Queensland University of Technology 2 George Street Brisbane QLD 4000 Australia; ^3^ Key Laboratory for Special Functional Materials of Ministry of Education National & Local Joint Engineering Research Center for High‐Efficiency Display and Lighting Technology School of Nanoscience and Materials Engineering Henan University Zhengzhou 450046 China; ^4^ School of Mechanical Medical and Process Engineering Queensland University of Technology 2 George Street Brisbane QLD 4000 Australia

**Keywords:** ammonia production, electrocatalyst, electrolyte, nitrate reduction reaction, strategies

## Abstract

The electrochemical reduction of nitrate for ammonia production not only offers a promising alternative to the traditional Haber–Bosch process, which requires high temperatures and pressures, but also provides an effective solution to the pollution caused by nitrogen‐enriched nutrients in drinking water and soil. Nitrate reduction is a complex multielectron, multiproton reaction, leading to multiple reaction pathways and numerous by‐products. Moreover, the product distribution and Faradaic efficiency are highly dependent on the applied potential, often resulting in competing reactions, such as the hydrogen evolution reaction, which increase energy consumption. Therefore, the development of low‐cost, highly active, highly selective, and scalable electrocatalysts for nitrate reduction is critical to advancing this field. This review highlights recent advances in nitrate reduction electrocatalysis, focusing on catalyst design strategies, reaction environments, and performance evaluation. It also compiles and analyzes a wide range of research examples in the field, discusses current challenges, and offers perspectives on future research directions. This review is aimed to serve as a guide for the rational design and development of nitrate reduction electrocatalysts and to accelerate progress in nitrogen cycle engineering.

## Graphical abstract

This review highlights the latest advances in electrocatalytic nitrate reduction to ammonia, with a focus on catalyst design strategies, electrolyte engineering, and performance evaluation.

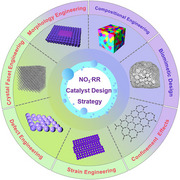

## 1 Introduction

The nitrogen cycle is a fundamental component of the global biogeochemical cycle and constitutes one of the primary material cycles within the biosphere.^[^ ^1^, ^2^, ^3^ ^]^ This cycle begins with atmospheric nitrogen entering the soil through the activity of microorganisms, where it is transformed into organic nitrogen compounds that can be utilized by plants and animals. Ultimately, nitrogen is returned to the atmosphere with the involvement of microorganisms. During the entire nitrogen cycle, a critical initial step is nitrogen fixation, namely, the conversion of inert atmospheric nitrogen (N_2_) into ammonia (NH_3_), which governs the input of bioavailable nitrogen into ecosystems.^[^ ^4^ ^]^ In natural systems, nitrogen fixation primarily occurs through nitrogen‐fixing organisms, such as rhizobia within leguminous root nodules, with minor contributions from abiotic phenomena, such as lightning. With the global population continuing to grow, the demand for artificial nitrogen fixation has increased significantly. Ammonia plays a vital role across numerous sectors, including agriculture (fertilizers), energy, defense, coatings, and explosives.^[^ ^5^, ^6^, ^7^, ^8^ ^]^ In the era of a low‐carbon economy, ammonia can serve as an indirect storage medium for hydrogen energy. This is attributed to several advantages of ammonia itself: i) Ammonia is carbon‐free and thus does not directly contribute to greenhouse gas emissions; ii) It has a high energy density, even higher than that of compressed air; iii) Ammonia can be easily liquefied at room temperature under moderate pressure; iv) The storage and transportation systems for ammonia are already well‐established. These advantages, along with its economic feasibility, make ammonia widely applicable in both the energy and agricultural sectors. Its applications include power generation, reciprocating engines, carbon dioxide removal via ammonia looping, fuel cells, gas turbines, propulsion technologies, and fertilizer production.^[^ ^9^, ^10^ ^]^ Currently, the Haber–Bosch process remains the dominant industrial method for ammonia synthesis. However, this method that relies on high temperature and pressure leads to significant energy usage and carbon dioxide emissions. As a result, the electrochemical nitrogen reduction reaction (NRR), which uses renewable energy to produce ammonia, has garnered considerable interest.^[^ ^11^ ^]^ Despite this, some major challenges, such as the competing hydrogen evolution reaction (HER) and the limitations of existing electrocatalysts, result in low ammonia yields and Faradaic efficiency, which is still far from meeting the demands of industrial‐scale production.^[^ ^12^, ^13^, ^14^ ^]^ Therefore, the development of cost‐effective and environmentally sustainable methods for ammonia synthesis is essential to address both global energy and environmental concerns and to sustain the nitrogen cycle.

**Figure 1:**
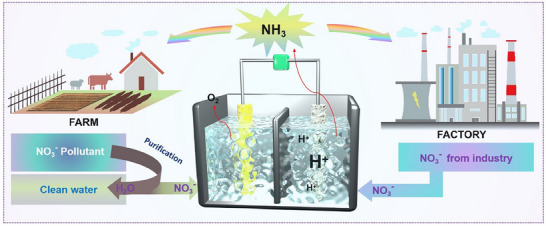
Schematic illustration of the fundamental characteristics and mechanistic pathways involved in nitrate reduction.

Given the high bond dissociation energy of the nitrogen triple bond (N≡N, 941 kJ mol^−1^) and the low solubility of nitrogen in water, it is crucial to identify alternative nitrogen sources with higher solubility and lower bond dissociation energy.^[^ ^15^ ^]^ Nitrate (NO_3_⁻) emerges as a promising candidate; its electrochemical reduction not only offers a pathway to ammonia synthesis but also contributes to mitigating nitrate pollution in wastewater (**Figure** **1**). Nitrate, a naturally existing ion, plays a vital role in the nitrogen cycle. Although most synthetic ammonia is used in nitrogen fertilizers, plants absorb only a fraction of the applied nitrogen^[^ ^16^ ^]^ Furthermore, the combustion of nitrogen‐containing industrial waste and fossil fuels generates significant nitrate pollutants.^[^ ^17^, ^18^ ^]^ Over time, excess nitrate can leach into surface and groundwater, which poses substantial risks to ecosystems and human health. Due to its high stability and solubility, nitrate is difficult to be removed from water, leading to eutrophication. Moreover, its reduction products, such as nitrite (NO_2_⁻), are even more harmful, causing adverse effects including goiter, congenital anomalies, methemoglobinemia in infants, and cancers.^[^ ^19^, ^20^, ^21^, ^22^ ^]^ According to the World Health Organization (WHO), the concentration of nitrate‐nitrogen in drinking water should be limited to no more than 10 mg L^−1^, yet many regions continue to exceed this threshold.^[^ ^23^, ^24^ ^]^

In the context of energy catalysis, the electrocatalytic reduction of nitrate (NO_3_^−^RR) to ammonia holds significant promise for addressing energy and environmental challenges. While the N═O bond in nitrate (204 kJ mol^−1^) has a lower dissociation energy than N≡N, the reduction of nitrate to ammonia proceeds through an eight‐electron transfer pathway, making the reaction pathway complex and prone to multiple by‐products, including NO_2_^−^, N_2_, N_2_O_4_, NO_2_, NO, and N_2_H_4_.^[^ ^25^, ^26^, ^27^ ^]^ The selectivity of this reaction is highly dependent on parameters, such as overpotential and current density. For typical nitrate reduction electrocatalysts with metallic active sites, the coupling between the valence orbitals of the metal and the lowest unoccupied molecular orbital of the NO_3_⁻ ion directly influences electron transfer between the catalyst and the nitrate ion. This interaction determines the potential‐determining step and the overpotential required for the onset of nitrate reduction reaction. For instance, cobalt‐based materials tend to reduce NO_3_⁻ to NH_3_ at more negative overpotentials.^[^ ^28^, ^29^ ^]^ Thus, the design of high‐efficiency, high‐selectivity, and low‐cost electrocatalysts is pivotal for enhancing the electroreduction of nitrate.

Recent studies have explored various strategies to improve ammonia production rates, Faradaic efficiency, nitrate conversion rates, and product selectivity. For example, crystal phase engineering can enhance the electrocatalytic performance for NO_3_^−^RR by tuning the atomic arrangement of catalysts. Previous studies have primarily focused on the regulation of conventional crystal phases. In recent years, nanomaterials with unconventional phases have also demonstrated superior catalytic activity for nitrate reduction compared to their traditional phase counterparts.^[^ ^30^, ^31^, ^32^, ^33^, ^34^, ^35^ ^]^ Researchers have investigated diverse catalyst systems and explored the mechanistic aspects of nitrate reduction through in situ characterization techniques. Additionally, studies have focused on the role of nonprecious metals and enzyme‐mimicking catalysts in facilitating nitrate reduction.^[^ ^25^, ^36^, ^37^, ^38^, ^39^, ^40^, ^41^, ^42^, ^43^ ^]^ In this review, a comprehensive summary of the recent advancements on NO_3_^−^RR is provided, focusing on catalyst engineering and electrolyte design. Particularly, several critical strategies are summarized, including morphology engineering, crystal facet engineering, strain engineering, defect engineering, alloying engineering, compositional engineering, doping engineering, confinement engineering, and biomimetic engineering. Collectively, these approaches offer valuable insights into enhancing catalytic activity and selectivity for NO_3_^−^RR. Hence, it provides timely and systematic guidance for future research.

## 2 General Principles for NO_3_^−^RR Catalyst Design

**Figure 2:**
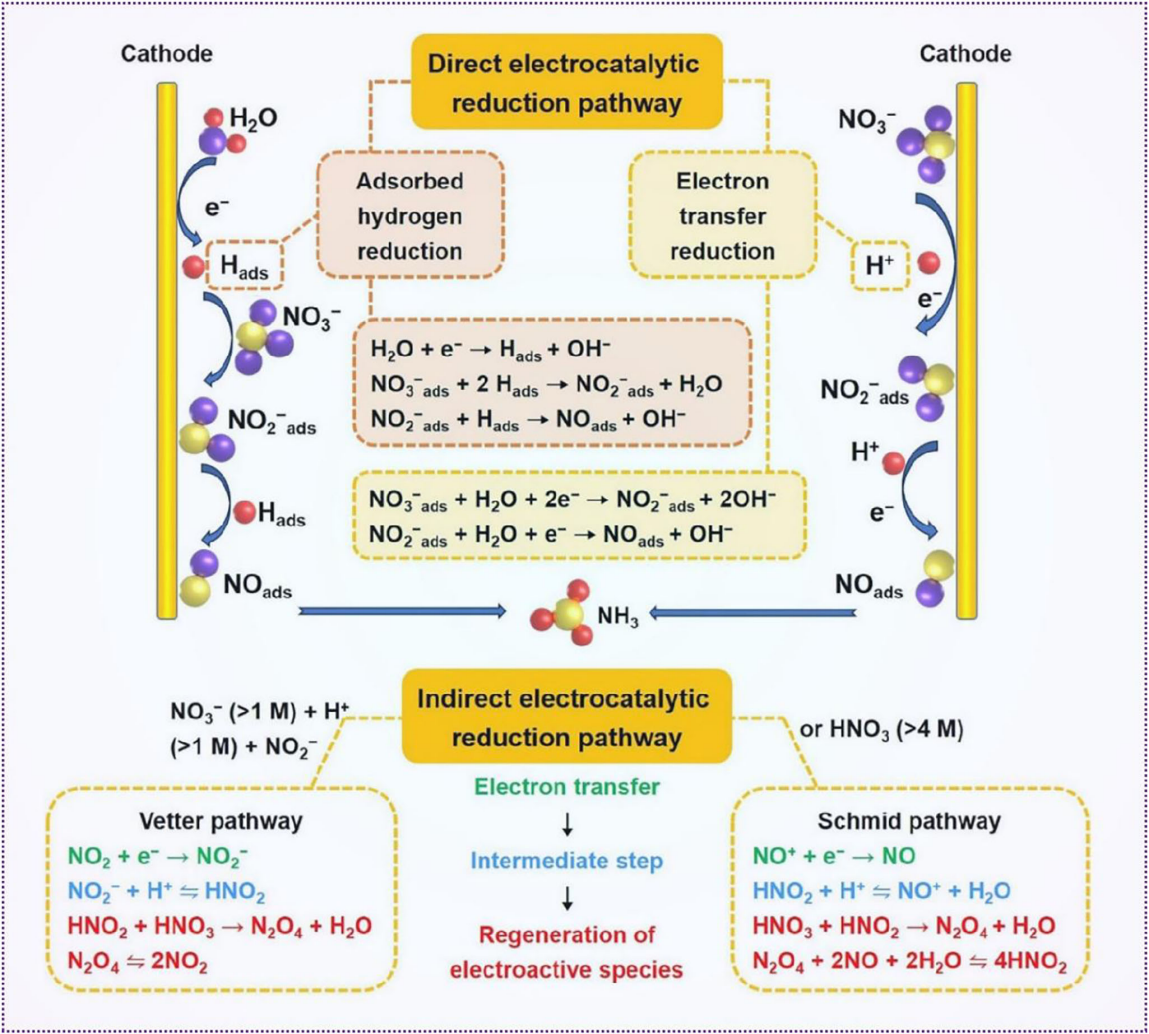
Reaction mechanism of nitrate reduction reaction. Reproduced with permission.^[^ ^44^ ^]^ Copyright 2024, Elsevier.

The NO_3_^−^RR is a complex multielectron process characterized by a wide range of oxidation states, abundant intermediates, and final products typically being N_2_ or NH_3_ (**Figure** **2**).^[^ ^44^, ^45^, ^46^ ^]^ The reaction pathway of NO_3_^−^RR is highly dependent on the concentration of nitrate ions and the pH of the solution, and it generally proceeds via two types of mechanisms: the direct and indirect pathways.^[^ ^47^, ^48^ ^]^ In most studies, the focus is on the direct pathway, which primarily involves two mechanisms: electron‐mediated reduction and atomic hydrogen‐mediated reduction. In the electron‐mediated process, the initial step of reducing NO_3_⁻ to NO_2_⁻ is considered the rate‐determining step. Subsequently, NO_2_⁻ is converted to adsorbed NO (NO_ads_), which is then further reduced to either NH_3_ or N_2_. The selectivity toward ammonia in this pathway remains a significant technical challenge. In the atomic hydrogen‐mediated reduction, active H_ads_ species generated by water electrolysis serve as strong reductants that reduce reaction intermediates. Here, adsorbed nitrogen (N_ads_) can either couple to form N_2_ or react with H_ads_ to generate NH_3_. This type of reduction typically occurs at lower overpotentials. In contrast, the indirect pathway does not involve direct electron transfer to NO_3_⁻ and tends to occur under high nitrate concentrations. In the Vetter pathway, NO_2_⁻ is protonated and ultimately reduced to NO_2_, while in the Schmid pathway, NO⁺ acts as the reactive species, which is further transformed into HNO_2_ during subsequent reactions.

**Figure 3:**
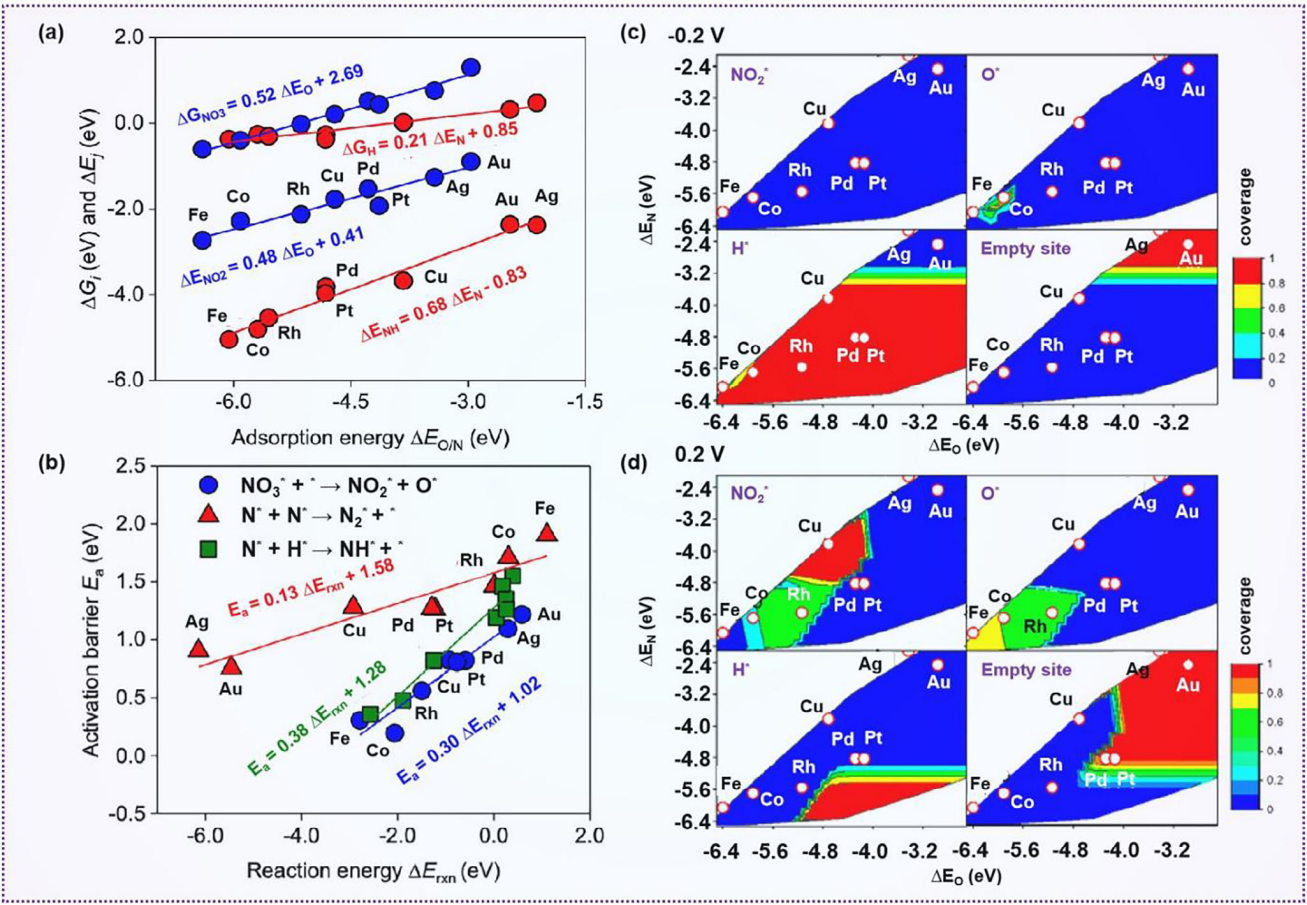
Variation in selectivity and catalytic activity of transition metals in nitrate electroreduction. a) Correlations between the Gibbs free energy of intermediate adsorption (ΔG) or electronic adsorption energy (ΔE) and the adsorption strength of nitrogen or oxygen species. b) Correlation of NO₃* dissociation, N₂* formation, and N* hydrogenation processes at 0 V versus RHE. c,d) Coverage distribution of key intermediates (NO_2_*, O*, and H*) for NO_3_RR at c) −0.2 V and d) 0.2 V. Reproduced with permission.^[^ ^49^ ^]^ Copyright 2019, American Chemical Society.

In the NO_3_^−^RR, the catalytic performance and the rate‐determining step are primarily governed by the adsorption strength of nitrogen and oxygen‐containing intermediates on the catalyst surface. Utilizing density functional theory (DFT) simulations and adsorption energy evaluations, various metal catalysts have been screened. Adsorption that is either too strong or too weak impedes the efficient progression of the reaction. For instance, Ag and Au exhibit the lowest activation barriers for N* recombination and subsequent hydrogenation steps involving N*, NH*, NH_2_*, O*, and OH*. In contrast, Fe and Co demonstrate the lowest barriers for dissociation of key intermediates, such as NO_3_*, NO_2_*, NO*, and N_2_O* (**Figure** **3**). These findings place the catalytic activity of Cu and Pt‐group metals for nitrate reduction at an intermediate level between these two extremes.^[^ ^49^ ^]^ In practical applications, the catalytic rate and rate‐determining step are strongly influenced by the applied potential, as surface coverage of adsorbed species (e.g., H and NO_3_^−^) can significantly alter the catalytic activity.

Pt‐group metals have attracted considerable attention for their high catalytic potential in nitrate reduction, particularly under acidic conditions. Notably, Pt electrodes are commonly used for NO_3_⁻RR in acidic media. Yang and co‐workers demonstrated that solution acidity plays a critical role in the reaction's efficiency.^[^ ^50^ ^]^ As pH increases, nitrate reduction performance declines, likely due to the pH dependence of proton‐coupled electron transfer (PCET) mechanisms. Among the Pt‐group metals, ruthenium (Ru) exhibits the highest electrocatalytic activity for nitrate reduction, as discussed by Dima et al.^[^ ^51^ ^]^ Li et al. synthesized Ru nanoclusters (≈2 nm in diameter) that achieved an ammonia yield of 5.56 mol g _cat_⁻¹ h⁻¹ at −0.8 V versus the reversible hydrogen electrode (RHE). This performance was attributed to lattice strain (≈12%) induced by subsurface oxygen modulation, which simultaneously suppresses HER and promotes NO_3_⁻RR.^[^ ^52^ ^]^

Despite the excellent selectivity and stability of noble metal catalysts, their high cost and limited availability present significant obstacles to widespread application. Consequently, increasing attention has been directed toward non‐noble metal catalysts, which offer advantages, such as low cost, abundant availability, and potential for large‐scale deployment. Among them, copper (Cu) stands out as a representative non‐noble metal catalyst for nitrate reduction. Electrochemical studies show that Cu maintains high current densities across the NO_3_⁻RR potential range, indicative of its favorable catalytic performance. Additionally, Cu exhibits the highest reaction kinetics for the rate‐determining step, making it particularly effective for NH₃ formation.^[^ ^53^ ^]^ Nonetheless, the relatively poor selectivity and limited stability of Cu catalyst remain significant challenges. Pure Cu electrodes are susceptible to oxidative dissolution and competitive adsorption of intermediates during the reaction, leading to catalyst deactivation or poisoning.^[^ ^54^ ^]^ Titanium (Ti) electrodes, by contrast, offer high selectivity over a broad range of operating conditions due to their intrinsic resistance to HER and strong corrosion resistance. McEnaney et al. systematically investigated the influence of pH, nitrate concentration, and applied potential on NO_3_⁻RR using Ti cathodes. Their findings suggest that elevated levels of both protons and nitrate ions are essential for achieving high selectivity.^[^ ^55^ ^]^ Ti also undergoes significant surface reconstruction during nitrate reduction, forming titanium hydride (TiH*_x_*, 0 < *x* ≤ 2). Liu et al. employed ex situ grazing‐incidence X‐ray diffraction and total electron yield X‐ray absorption spectroscopy to demonstrate that TiH₂‐enriched surfaces enhance both the applied potential window and operational stability for nitrate reduction.^[^ ^56^ ^]^

Significant efforts have been dedicated to developing electrocatalysts for nitrate reduction with enhanced activity, high Faradaic efficiency, and improved selectivity toward ammonia. Various strategies have been proposed and implemented to achieve these goals, including tailoring catalyst morphology and crystal facets, introducing secondary metals to form alloys or heterostructures, engineering surface vacancies, applying surface modifications, and increasing the density of active sites to improve catalyst utilization.

From the basic characterization of catalyst design to its performance in NO_3_^−^RR, this follows a conventional experimental pathway. However, for certain catalyst systems, a thorough theoretical explanation should be provided. For example, Cu‐doped Co_3_O_4_ shows a reduced overpotential for NO_3_^−^RR and improved ammonia yield compared to pristine Co_3_O_4_. According to the molecular orbital theory, Cu doping leads to an upward shift of the highest occupied state (HOS) of Co_3_O_4_, thereby narrowing the energy gap between the HOS of Co_3_O_4_ and the lowest unoccupied molecular orbital (LUMO) of NO_3_⁻. This reduction in energy barrier facilitates the electron transfer from Co_3_O_4_ to NO_3_⁻, effectively lowering the overpotential required to initiate the reaction and endowing the catalyst with superior nitrate‐to‐ammonia conversion performance.^[^ ^57^ ^]^ In general, electrocatalysts exhibit adsorption–energy scaling relationships, which can inherently limit their catalytic performance. To overcome these limitations, it is essential to develop new theoretical design principles that break such scaling relations. Gao et al. employed Bayesian chemical adsorption theory (Bayeschem), a machine learning‐based approach, to uncover the origin of the linear scaling relationship between *NO_3_ and *N adsorption energies on metal surfaces. They identified an intriguing mechanism in which site‐specific Pauli repulsion between the metal d‐states and the frontier orbitals of adsorbates can be utilized to break the scaling relation. Using this insight, they synthesized ordered intermetallic B2 CuPd nanocubes with (100) crystal facets via a colloidal method, achieving a Faradaic efficiency of 92.5% in alkaline media. Theoretical analysis revealed that although the upward shift of the Cu *d*‐band center favors *NO_3_ adsorption, the dominant Pauli repulsion from the subsurface Pd d‐orbitals destabilizes the hollow‐site *N intermediate. This destabilization facilitates the protonation of nitrogen‐containing intermediates, thereby enhancing the formation of NH_3_.^[^ ^58^ ^]^ For nitrate reduction electrocatalysts, the position of the *d*‐band center relative to the Fermi level plays a crucial role in determining catalytic activity and selectivity. According to the *d*‐band center theory, a higher *d*‐band center leads to stronger adsorption of reaction intermediates, while a lower *d*‐band center results in weaker adsorption. If the *d*‐band center is too high, it may cause catalyst poisoning due to overly strong binding. Conversely, if the *d*‐band center is too low, the adsorption may be too weak to effectively activate nitrate molecules. Therefore, an optimal *d*‐band center is essential to balance the adsorption and desorption of key intermediates, reduce the overpotential, and enhance ammonia selectivity and Faradaic efficiency. For example, Yao et al. doped Ru into a Ni‐based metal–organic framework, thereby tuning the *d*‐band center of adjacent Ni sites upshift.^[^ ^59^ ^]^ This adjustment optimized the adsorption strength of nitrogen‐containing intermediates and significantly improved the performance of the nitrate reduction reaction.

## 3 Engineering for Efficient NO_3_^−^RR Catalyst

**Figure 4:**
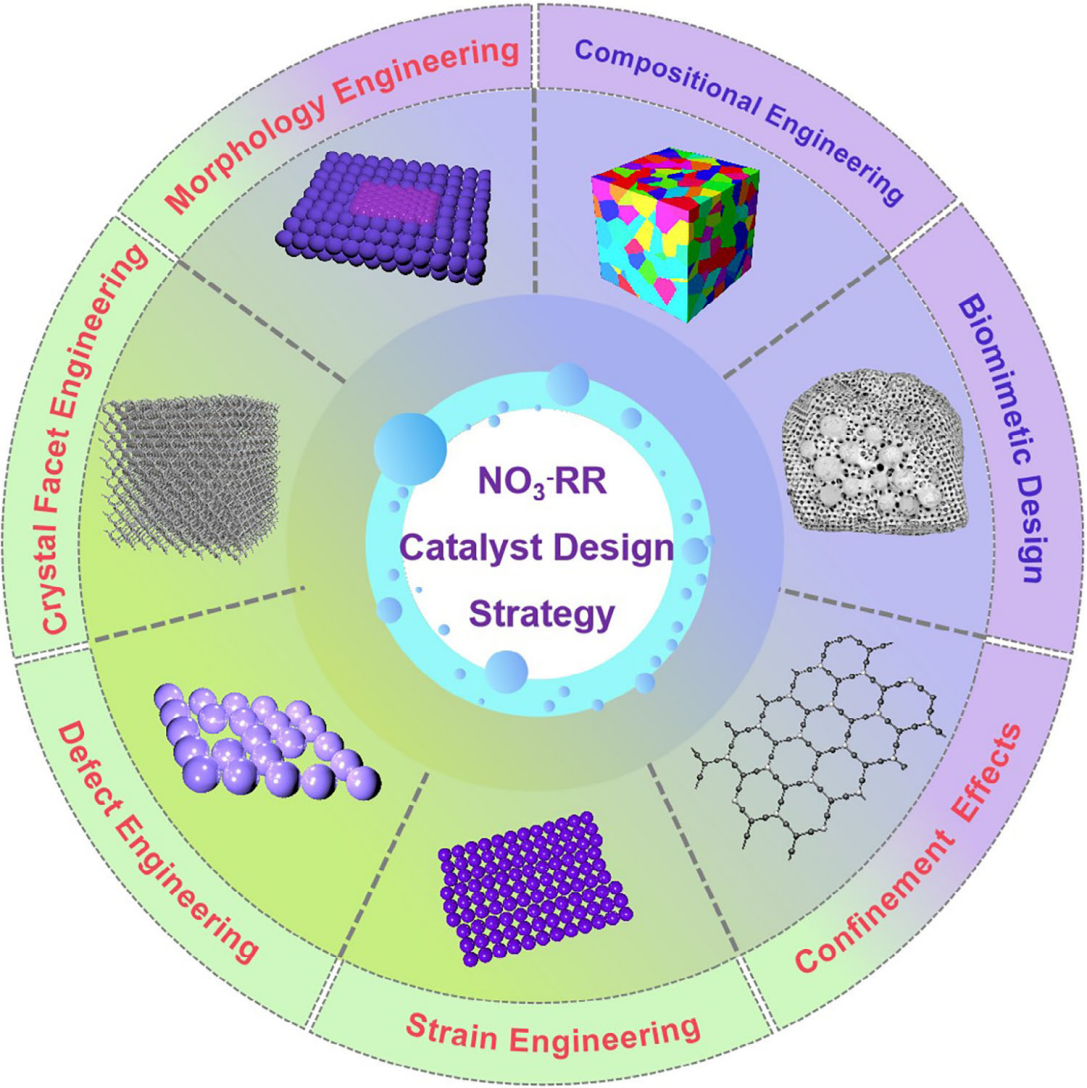
A summary of various engineering strategies to modulate the nitrate reduction performance of electrocatalysts.

Given the growing scientific and practical interest in NO_3_⁻RR, it is imperative to comprehensively summarize recent advancements and identify the key challenges that remain. As summarized in **Figure** **4**, this section outlines and discusses several critical strategies for catalyst design and regulation, including morphology engineering, crystal facet engineering, strain engineering, defect engineering, compositional engineering, confinement engineering, and biomimetic engineering.

### 3.1 Morphology Engineering

The chemical properties of nanocrystals are intrinsically linked to their structural morphology, as both size and shape significantly influence their physicochemical behavior.^[^ ^60^, ^61^, ^62^, ^63^, ^64^, ^65^ ^]^ Similar to other 2D nanomaterials, nanotube structures possess a high specific surface area and low density, providing a high density of active sites owing to the abundance of unsaturated metal atoms on the surface. The development and application of morphology‐controlled nanoparticles have had a transformative impact on the field of electrocatalysis. Not only have these materials substantially enhanced catalytic activity for various electrochemical reactions, but they have also provided deeper insights into how nanoscale morphology and surface structure influence electrochemical NO₃⁻RR activity.

Generally, electrocatalytic performance is highly structure‐dependent, and various synthetic strategies have been employed to rationally engineer Cu‐based structures to optimize their nitrate reduction capabilities.^[^ ^69^, ^70^ ^]^ For instance, Wang et al. developed a novel 3D Cu nanobelt cathode that demonstrated exceptional performance for electrochemical nitrate reduction. After 60 min of reaction time, this material achieved 100% nitrate removal, significantly outperforming conventional Cu foam electrodes, which showed only 2.6% removal under identical conditions.⁴^3^ This enhancement is attributed to two main factors: the large specific surface area of the 3D Cu nanobelts, which promotes rapid mass transfer and accelerates electrochemical kinetics, and the preferential interaction between the nanobelt surface and nitrate ions, as opposed to dissolved oxygen, which minimizes undesirable H_2_O_2_ formation.^[^ ^71^ ^]^ In another study, Li et al. prepared Cu nanotubes featuring sheet‐like surface structures by first chemically oxidizing Cu nanowires, subsequently undergoing in situ electrochemical reduction. Compared to the original Cu nanowires, the Cu nanotubes exhibited a significantly higher nitrate reduction rate (778.6 µg h^−1^ mg^−1^) and improved selectivity (86.2%) for ammonia production. The superior performance was ascribed to their unique morphology, which not only facilitated mass transport but also conferred increased resistance to dissolution and minimized catalyst aggregation.^[^ ^72^ ^]^

**Figure 5:**
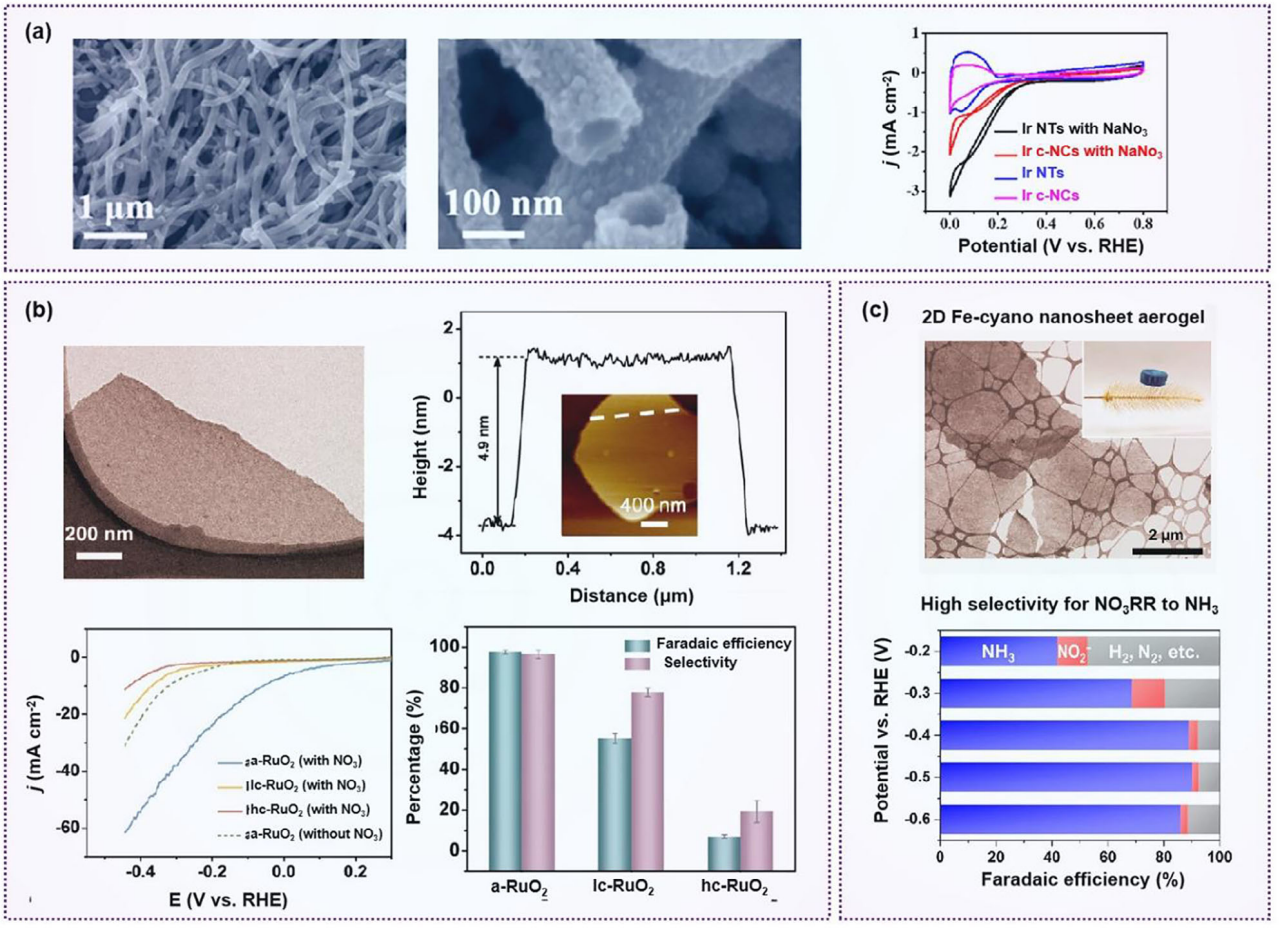
Morphology effect on nitrate reduction. a) Ir nanotubes (Ir NTs) and the comparison with Ir nanocrystals (Ir NCs) in nitrate reduction. Reproduced with permission.^[^ ^66^ ^]^ Copyright 2020, American Chemical Society. b) 2D RuO_2_ nanosheets and the nitrate reduction performance in comparison with amorphous RuO_2_ nanosheets grown on a carbon paper substrate (a‐RuO_2_), low crystallinity (Ic‐RuO_2_), high crystallinity (hc‐RuO_2_). Reproduced with permission.^[^ ^67^ ^]^ Copyright 2022, Wiley‐VCH GmbH. c) 2D Fe‐cyano nanosheets (Fe‐cyano NSs) in NO_3_ reduction to NH_3_ and the selectivity at various potentials. Reproduced with permission.^[^ ^68^ ^]^ Copyright 2021, American Chemical Society.

Beyond Cu‐based materials, hollow Ir nanotubes have also demonstrated outstanding performance for nitrate reduction, achieving an NH₃ production rate of 921 µg h⁻¹ mg_cat_⁻¹ and a Faradaic efficiency of 84.7% at 0.06 V (**Figure** **5**a). In comparison to commercial Ir nanocrystals (Ir c‐NCs), the Ir nanotubes (Ir NTs) exhibit a Faradaic efficiency and nitrate reduction yield of merely 53.6% and 334 µg h⁻^1^ mg_cat_⁻^1^ at 0.06 V. This is mainly due to the high specific surface area, rough porous surface, and abundant defects of the prepared 1D Ir NTs.^[^ ^66^ ^]^ Similarly, 2D RuO₂ nanosheets synthesized via a molten salt method exhibited excellent selectivity (96.42%) and high Faradaic efficiency (97.46%) for ammonia production from nitrate (Figure 5b). They used 2D RuO_2_ nanosheets as the research subject and tuned the crystallinity of the material by controlling the temperature. They found that the amorphous 2D structure featured a disordered atomic arrangement and abundant oxygen vacancies, which facilitated the formation of key NH₃* intermediates, thus improving the selectivity and Faradaic efficiency of the nitrate reduction process.^[^ ^67^ ^]^

Metal–organic frameworks (MOFs) have attracted growing attention as electrocatalysts due to their tunable compositions and highly porous structures. Among them, metal‐cyano coordination polymers, a class of 3D inorganic polymeric gels composed of polymetallic units bridged by cyano (*─*CN⁻) groups, exhibit porous, compositionally adaptable frameworks. The presence of hydrophilic groups and surface defects enhances their electrocatalytic activity. Fang et al. synthesized porous 2D Fe‐cyano nanosheets, which exhibited high selectivity, yield, and stability for nitrate reduction. These characteristics stem from the strong adsorption of nitrate by metallic iron, the high surface area, and the confined interconnection structure of the nanosheets (Figure 5c).^[^ ^68^ ^]^ Zirconium‐based MOFs (Zr‐MOFs) also show promise due to their inherent conductivity and porosity. Jiang et al. utilized in situ reduction techniques to grow precious metal nanodots (Pd, Ag, Au) on Zr‐MOF substrates. The MOF structure incorporated redox‐reversible tetrathiafulvalene linkers, which further enhanced the electrocatalytic properties. Among the tested materials, Pd‐NDs/Zr‐MOF demonstrated the highest catalytic performance for nitrate reduction.^[^ ^73^ ^]^

Besides, an one of earth‐abundant transition metal oxides, spinel‐type oxides (AB_2_O_4_) are particularly attractive for NO_3_⁻RR due to their superior electronic conductivity, flexible ion arrangements, multivalent structures, and structural versatility.^[^ ^74^ ^]^ Notably, NiCo_2_O_4_ nanowires and 3D flower‐like ZnCo_2_O_4_ catalysts demonstrated higher Faradaic efficiency and ammonia production rates compared to Co_3_O_4_.^[^ ^75^, ^76^ ^]^ Specifically, under alkaline conditions at −0.3 V, NiCo_2_O_4_ achieves a Faradaic efficiency of up to 99.0%, and at −0.6 V, it reaches an ammonia yield rate of 973.2 µmol h^−1^ cm^−2^ which outperforms Co_3_O_4_ under the same conditions. The introduction of Zn induces electron transfer that renders Co in an electron‐deficient state, thereby lowering the energy barrier for *NO_2_ intermediate formation and suppressing the hydrogen evolution reaction. ZnCo_2_O_4_ achieves an ammonia yield rate of 2100 µg mg⁻^1^ h⁻^1^, ≈2.0 times higher than that of Co_3_O_4_, with a Faradaic efficiency of around 95.4%.

As expected, morphology engineering of electrocatalysts holds a key position in determining the ammonia yield and Faradaic efficiency of nitrate electroreduction. Morphological control can be achieved across different scales and in dimensionality, ranging from 0D to 3D structures. For instance, 1D nanocatalysts typically exhibit efficient electron transport; 2D nanomaterials have distinct surface electronic states; and 3D architectures often promote mass diffusion due to their porous structures. At the nanoscale, morphology can influence the local electronic structure, potentially leading to lattice distortion and other beneficial effects. Controlling the shape and size of nanoparticles may also influence adsorption/desorption behavior, wettability, and mass transport. Therefore, the rational design and development of advanced electrocatalysts for nitrate reduction remain essential. Given that nitrate reduction is a complex multielectron process with multiple possible products, the design of structured electrodes tailored for specific target products is equally important. This can involve the incorporation of catalysts with specific geometric arrangements or ordered structures. Moreover, the morphology of the catalyst is closely related to its long‐term stability. Thus, considerations of structural collapse and aggregation during extended operation should also be integrated into the design strategy.

### 3.2 Crystal Facet Engineering

Facet engineering has emerged as a highly promising strategy for optimizing the distribution of specific crystal planes on a catalyst's surface. Due to anisotropic effects, crystal facets with different orientations can significantly influence physical and chemical properties. In electrocatalysis, polycrystalline catalysts often exhibit facet‐dependent behavior, where differences in geometric structure, redox‐active sites, surface electronic distribution, and built‐in electric fields, which arises from the orientation of exposed facets, play crucial roles in determining catalytic activity. These properties directly affect the adsorption energy of key reaction intermediates, thus influencing both the electrochemical activity and selectivity in nitrate reduction. Highly active facets typically expose a larger number of reactive sites, which enhances the intrinsic catalytic activity and lowers the activation energy for the reaction. Therefore, precise control over the exposure of specific facets is considered an effective approach to improve catalytic efficiency, reduce reaction energy barriers, and accelerate reaction rates.^[^ ^77^ ^]^

**Figure 6:**
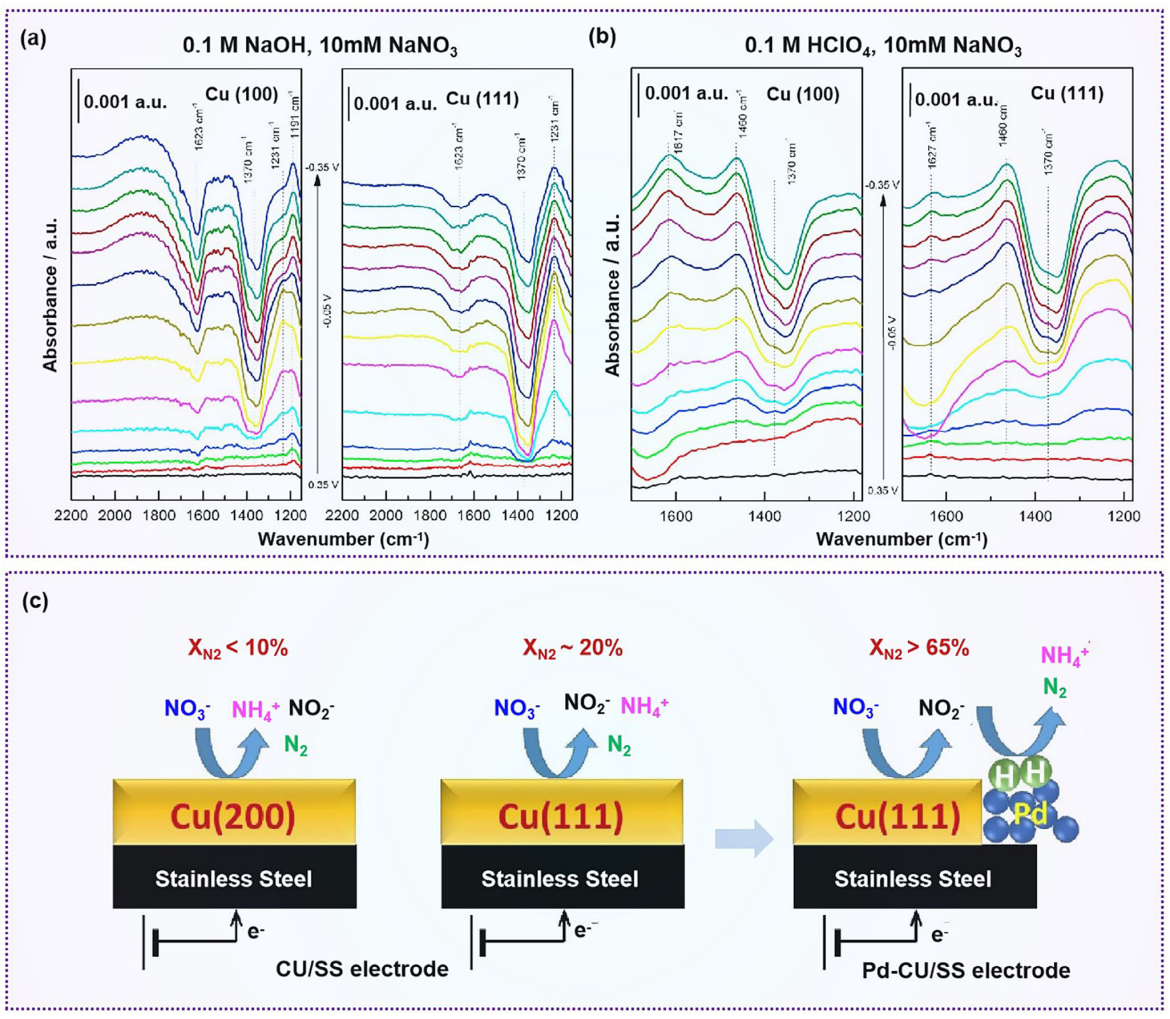
Crystal facet engineering on nitrate reduction. a,b) Absorbance spectra for the reduction of 10 mm NaNO_3_ on Cu (100) and Cu (111) electrode. Reproduced with permission.^[^ ^78^ ^]^ Copyright 2017, Elsevier. c) Selectivity of facet orientation of Cu for NO₃⁻RR. Reproduced with permission.^[^ ^79^ ^]^ Copyright 2020, Elsevier.

For instance, Pérez‐Gallent et al. investigated the nitrate reduction performance on Cu(100) and Cu(111) surfaces in both alkaline and acidic electrolytes using a combination of electrochemical techniques, including cyclic voltammetry and in situ characterization (**Figure** **6**a,b). In alkaline media, the onset potential for nitrate reduction on Cu(100) was +0.1 V, 50 mV earlier than on Cu(111), indicating that Cu(100) is more active under alkaline conditions. The product distribution was also found to be pH‐dependent: NO and NH₃ were favored in acidic media, while hydroxylamine was predominantly formed under alkaline conditions.^[^ ^78^ ^]^

Shih et al. further demonstrated the ability to modulate nitrate reduction selectivity by controlling the ratio of exposed crystal facets (Figure 6c). Surfactants such as cetyltrimethylammonium chloride, polydiallyldimethylammonium chloride, and benzethonium chloride were employed to tune the growth orientation of Cu nanocrystals. A higher proportion of Cu(111) facets significantly enhanced N₂ selectivity, suggesting that Cu(111) plays a key role in directing product outcomes. Upon alloying with Pd, an optimal Pd:Cu ratio of 0.27:0.73 was found to enhance N_2_ yield up to 65%, with the Cu(111) facet maintaining its favorable influence on N₂ selectivity.^[^ ^79^ ^]^

Rhodium (Rh) has also shown superior activity compared to Pt for NO_3_⁻RR, making it an attractive candidate for catalyst development. To elucidate the reaction mechanism, Guo et al. employed DFT calculations to model the nitrate reduction process on Rh(111), Rh(110), and Rh(100) surfaces in alkaline media. These calculations evaluated the energetics of key intermediates, including NO_2_⁻, NO, N_2_O, N_2_, and NH_3_, and revealed that Rh(100) exhibits the highest activity and selectivity toward NH₃ production within the potential window of −0.57 to 0.15 V. This facet also demonstrated the lowest energy barrier for the rate‐determining step (*N → *NH) and a low nitrate surface coverage, which suppresses undesired nitrogen formation. Rh(110) showed broader potential‐dependent activity toward ammonia below 0.06 V, while Rh(111) exhibited negligible NH₃ formation due to its high activation barrier, despite an onset potential of −0.07 V. These findings were experimentally validated using commercial Rh/C catalysts, with observed trends in ammonia yield and Faradaic efficiency aligning well with the theoretical predictions.^[^ ^80^ ^]^

In summary, crystal facet engineering is considered a highly effective strategy to enhance the performance of catalysts for nitrate reduction. The exposed crystal facets directly determine the arrangement and orientation of surface atoms, which in turn influence the adsorption and activation of reactants and intermediates—factors that fundamentally govern catalytic activity.^[^ ^81^ ^]^ However, synthesizing catalysts with high‐index facets to expose active planes without altering the composition remains a significant challenge. Typically, precise control over crystal facets requires a deep understanding of crystal growth behavior, as the exposure of specific facets is governed by their relative growth rates. Therefore, the key to tuning facets lies in manipulating the crystal growth kinetics. Surfactants can be employed to control the growth rate of facets, but their strong adsorption may also hinder the catalytic activity of those facets. Additionally, the introduction of other metals can modulate the surface energy of specific facets. Crystal facet engineering can be synergistically combined with other strategies, such as alloying and doping, to further improve nitrate reduction performance.

### 3.3 Strain Engineering

The binding strength of the adsorbate to the catalytic site is essential in determining the overall reaction energy barrier. This surface chemistry is intimately linked to the electronic structure of the catalyst. Consequently, tuning the electronic structure of catalysts is essential in optimizing various catalytic processes, including nitrate reduction. Among several strategies, adjusting lattice strain is particularly powerful because it can influence the atomic spacing and electron interactions within the catalyst. Strain effects arise from bond length variations within the lattice or mismatches between atomic layers, which can alter the catalyst's electronic properties. These strain‐induced changes can, in turn, affect the adsorption behavior of reactants, intermediates, and products, making strain engineering a key tool for enhancing catalytic performance, particularly in nitrate reduction.

**Figure 7:**
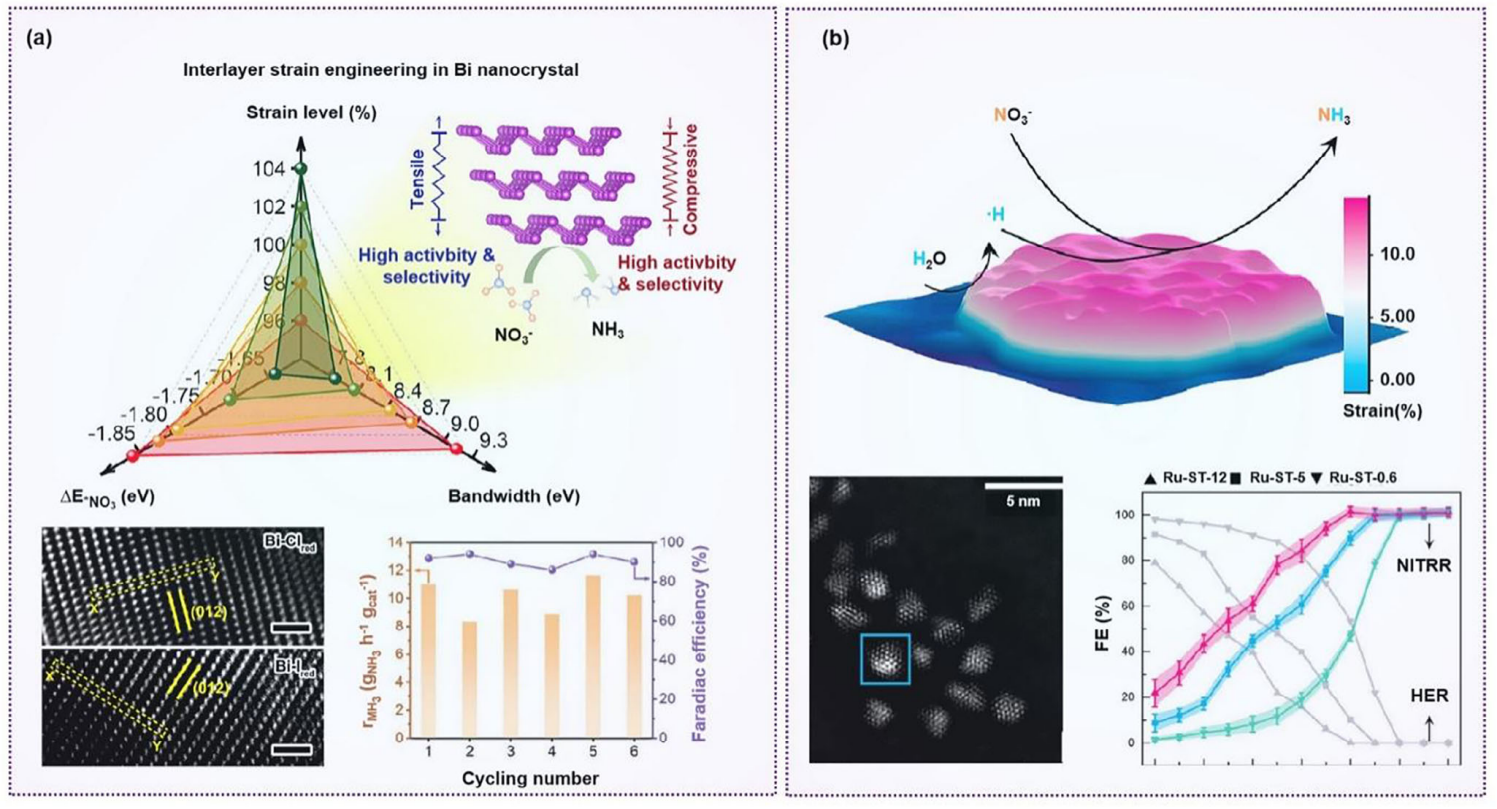
Strain engineering on nitrate reduction. a) A spider‐like chart illustrating the relationship among interlayer strain level, Bi‐6*p* bandwidth and adsorption energy of *NO_3_^−^, TEM images of Bi‐X_red_ catalyst, and cycling tests. Reproduced with permission.^[^ ^82^ ^]^ Copyright 2022, American Chemical Society. b) Faradaic efficiencies of NO₃⁻RR and HER of different Ru‐based core/shell clusters. Reproduced with permission.^[^ ^52^ ^]^ Copyright 2020, American Chemical Society.

Zhang et al. used theoretical calculations to demonstrate that interlayer strain compression in the Bi lattice matrix leads to the delocalization of its 6*p* electron band, thereby enhancing interactions with nitrogen‐containing species. This strain‐induced chemical binding facilitates the conversion of nitrate to ammonia and improves the activity and stability of nitrate reduction. They synthesized Bi catalysts with different interlayer distances by electroreduction of BiOX and found that the Bi−Cl_red_ catalyst exhibited superior selectivity and electrocatalytic activity for nitrate reduction. This was mainly attributed to the compressive lattice strain (**Figure** **7**a).^[^ ^82^ ^]^

Single‐atom catalysts are particularly advantageous due to their ultrahigh metal utilization efficiency. Huang et al. used a single‐atom Cu*─*N*─*C catalyst as a model system, wherein they precisely modulated the electronic structure of Cu by introducing B atoms into the first and second coordination shells. This modification induced two opposing effects: the first‐shell B coordination created tensile strain in the Cu*─*N bond and reduced the Cu valence state, while the second‐shell B coordination induced compressive strain and increased the Cu valence state. The position and number of B atoms were adjusted to flexibly and precisely modulate the electronic structure of Cu, leading to a significant improvement in electrocatalytic nitrate reduction performance. The Cu*─*N_4_B_2_ catalyst optimized the adsorption strength of the *NO intermediate, thereby lowering the energy barriers for nitrate reduction and water dissociation, achieving an impressive Faradaic efficiency of 98.2%, surpassing most Cu single‐atom catalysts.^[^ ^83^ ^]^ For specific single‐atom catalysts, strain‐induced changes in metal–ligand bond lengths and angles can disrupt geometric symmetry and cause perturbations in the electronic structure. Generally, tensile strain leads to a downward shift of the d‐band center of the single metal atom, while compressive strain results in an upward shift. These shifts significantly influence the adsorption energy and activation barriers. Strain can effectively modulate the position of the *d*‐band center, thereby enhancing or weakening the interaction between the catalyst and adsorbates. Liu et al. prepared Pd single atoms suspended on unsaturated bonds formed between CuO atomic layers due to dislocations via a low‐temperature treatment. The Pd single atoms located at the dislocation sites were subjected to shear stress and dynamic interactions with the support, which facilitated the conversion of nitrate to ammonia. At −0.5 V, the ammonia yield reached 4.2 mol  g_cat_^−1^ h^−1^.^[^ ^84^ ^]^

Li et al. prepared strain‐engineered electrocatalysts by introducing subsurface oxygen into Ru nanoclusters, in which ruthenium oxychloride was synthesized via a sol–gel method and was then electrochemically reduced to obtain strained Ru nanoclusters that retained subsurface oxygen while completely removing chlorine. These 2 nm Ru nanoclusters exhibited an NH_3_ production rate of 5.56 mol g_at_^−1^ h⁻¹ at −0.8 V versus RHE. Experimental results indicated that tuning the subsurface oxygen concentration induced a 12% tensile lattice strain, which effectively suppressed the HER while promoting nitrate reduction (Figure 7b).^[^ ^52^ ^]^

Titanium dioxide (TiO_2_) has demonstrated a relatively high catalytic effect for nitrate reduction. The introduction of heteroatom doping, such as Pd atoms, into TiO_2_ can widen the TiO_2_ lattice and reduce the surface work function of the catalyst. This alteration weakens the adsorption of intermediate products during the nitrate reduction process, leading to significantly improved Faradaic efficiency and nitrate conversion.^[^ ^85^ ^]^

Strain engineering has emerged as a powerful strategy for modulating the adsorption and activation of reactants and intermediates during NO_3_^−^RR. By applying lattice strain, either tensile or compressive, through some techniques, such as lattice mismatch, heterostructure construction, atomic doping, or nanostructuring, the atomic arrangement and electronic density of states at the catalyst surface can be precisely adjusted. These modifications can shift the *d*‐band center of transition metals or influence orbital hybridization, thereby tuning the adsorption energy of intermediates and improving both activity and selectivity.

Despite its promise, the practical implementation of strain engineering faces several critical challenges. First, accurately controlling the location, direction, and intensity of strain within a catalyst material during synthesis is highly complex. Strain distribution is often inhomogeneous and strongly dependent on factors, such as particle morphology, substrate interaction, and thermal treatment history. As a result, strain states are frequently estimated through theoretical simulations, but are difficult to confirm or reproduce experimentally with high fidelity. Second, the inherent metastability of strain‐engineered catalysts under reaction conditions poses significant limitations. Prolonged electrochemical operation can induce relaxation, reconstruction, or phase transformation of strained structures, leading to performance degradation and reduced long‐term stability and selectivity. Third, despite advances in in situ and operando techniques, such as XAS, TEM, and Raman spectroscopy, real‐time characterization of strain evolution at the atomic level during nitrate reduction is still technically demanding. Limitations in spatial and temporal resolution hinder the direct observation of local strain effects and their correlation with reaction intermediates and kinetics. To overcome these limitations, future progress will require the integration of multiple approaches. On the synthesis side, advanced techniques such as atomic layer deposition, strain‐tunable substrates, or template‐assisted growth could provide more precise control over strain. On the characterization side, combining high‐resolution operando tools with machine‐learning‐based analysis could offer deeper insights into the strain‐performance relationship. Furthermore, the coupling of theoretical modeling with experimental validation will be essential to guide rational design and to bridge the gap between predicted strain effects and real‐world catalytic behaviors. In summary, although strain engineering offers great promise for improving nitrate reduction, overcoming its key challenges is essential for practical use.

### 3.4 Defect Engineering

In general, structural defects are effective in regulating catalytic performance. These defects disrupt or even break the periodic crystal lattice, leading to a redistribution of the chemical and electronic properties of nanomaterials. Structurally, defects can be categorized as bulk or surface defects, depending on their spatial location. Given that electrocatalytic reactions predominantly occur on catalyst surfaces, surface defects, such as point, line, and planar defects, are of particular importance. Among these, point defects are the most extensively studied, as they involve deviations from the regular atomic arrangement at specific lattice sites, significantly modulating the electronic structure and surface/interface properties of the catalyst.

Doping defects arise when heteroatoms replace host atoms or occupy interstitial sites within the lattice, while vacancies, either from missing atoms or extraneous atoms in normally vacant positions, can further disrupt the structure and alter electronic properties. By precisely tuning the type, concentration, and spatial distribution of such defects, researchers can effectively control the adsorption energy of intermediates and reaction pathways during NO_3_^−^RR.

**Figure 8:**
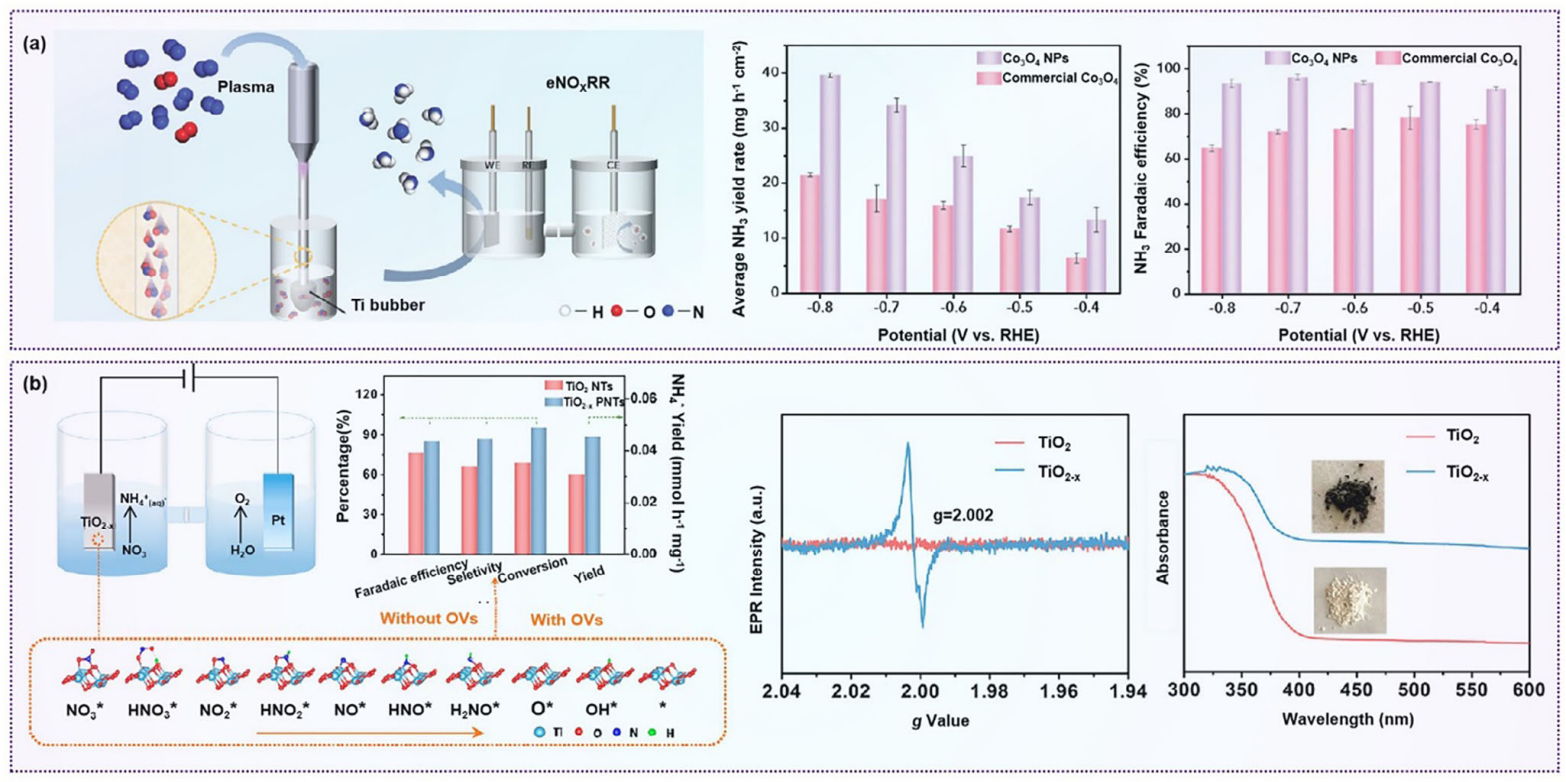
Defect engineering on nitrate reduction. a) Schematic illustration of the nitrogen fixation system, the yield rate of NH_3,_ and Faradaic efficiency using Co_3_O_4_ in plasma‐generated NO*_x_* ^−^ in NaOH solution. Reproduced with permission.^[^ ^86^ ^]^ Copyright 2022, Wiley‐VCH. b) Schematic illustration of NO₃^−^RR by TiO_2−_ *_x_* nanotubes, electron paramagnetic resonance (EPR) spectra, and ultraviolet–visible (UV–vis) spectra. Reproduced with permission.^[^ ^87^ ^]^ Copyright 2020, American Chemical Society.

While most studies focus on the direct reduction of nitrate in solution, Meng et al. demonstrated a two‐step process converting atmospheric nitrogen into ammonia. In the first step, nitrogen was converted into NO*x* in air and then dissolved into NO*x*⁻ in solution. The second step involved electrochemical conversion to ammonia. For this process, they utilized oxygen‐vacancy‐rich Co_3_O_4_ nanoparticles as electrocatalysts, which significantly enhanced nitrogen fixation performance (**Figure** **8**a). This catalyst achieved 96.08% Faradaic efficiency and an NH₃ production rate of 39.60 mg h^−1^ cm^−2^. The oxygen vacancies promoted the activation of Co sites, enhanced the hydrogenation and adsorption of NO*x*⁻ species, and simultaneously suppressed competing hydrogen evolution reactions.^[^ ^86^ ^]^

Gong et al. improved NO₃RR activity by modifying Cu₂O catalysts using argon plasma treatment. Their combined use of in situ diffuse reflectance infrared Fourier transform spectroscopy (DRIFTS), synchrotron‐based X‐ray absorption spectroscopy, and DFT calculations revealed that Ar plasma effectively promoted the formation of hydroxyl groups and surface oxygen vacancies. These changes enhanced nitrate adsorption and proton transfer, ultimately boosting Faradaic efficiency and ammonia production rates.^[^ ^88^ ^]^

Similarly, TiO_2_₋*x* nanotubes with oxygen vacancies have shown promising performance in NO_3_^−^RR. During the reaction, oxygen atoms from the nitrate molecules fill the vacancies in TiO_3_₋*x*, thereby weakening the N*─*O bond. This results in reduced by‐product formation and improved Faradaic efficiency and ammonia yield (Figure 8b).^[^ ^87^ ^]^ Heteroatom doping can also introduce defect sites. For instance, Xu et al. employed a hierarchical‐defect engineering strategy to synthesize Pd‐Cu_2_O catalysts with controlled cavity structures and oxygen vacancies. Their Pd‐Cu_2_O CEO catalyst, containing only 2.93 at% Pd, demonstrated excellent nitrate adsorption and subsequent reduction capabilities. It achieved an NH₃ yield of 925.11 µg h^−1^ mg_at_^−1^, an NH_3_ selectivity of 95.31%, and a Faradaic efficiency of 96.56%.^[^ ^89^ ^]^

The crystallinity of catalysts also significantly influences their catalytic behavior. Wang et al. prepared three different RuO_2_ forms, namely, amorphous, low‐crystalline, and highly crystalline, and evaluated their NO_3_RR performance. Amorphous RuO_2_ outperformed the others due to its disordered atomic arrangement, which generated a high density of oxygen vacancies. These vacancies promoted the formation of NH_3_* intermediates and modulated hydrogen affinity, thereby enhancing both selectivity and Faradaic efficiency.^[^ ^67^ ^]^ Amorphous Cu catalysts have also been employed for the electrosynthesis of NH_3_ from nitrate. This amorphous structure is rich in low‐coordinated atoms and defects, which provide more active sites and enhanced electrode–electrolyte interfaces. Compared to the crystalline Cu, the amorphous Cu exhibits an upward‐shifted *d*‐band center closer to the Fermi level, resulting in stronger adsorption of reaction intermediates during the nitrate reduction process.^[^ ^90^ ^]^

Given their low cost, tunable electronic structure, and excellent durability, nonmetallic materials have also attracted considerable attention for efficient NO_3_^−^RR. Carbon‐based materials, in particular, have been widely studied, including conductive diamond, carbides, and graphene. Defect‐rich graphene has shown a more positive onset potential than Pt in acidic nitrate solutions. Moreover, the presence of nitrite accelerates nitrate reduction on graphene, suggesting that nitrite reduction is the rate‐determining step.^[^ ^91^ ^]^ Beyond graphene, Zhu et al. employed in situ electrochemical activation to create defect‐ and heteroatom‐rich carbon fiber paper. These modifications improved NO₃RR performance by enhancing surface wettability and reactivity at defect sites.^[^ ^92^ ^]^ Additionally, Huang et al. utilized nitrogen vacancies to tune the properties of graphitic carbon nitride (g‐C_3_N_4_). Introducing a moderate amount of nitrogen vacancies generated new electronic states near the Fermi level, thereby improving conductivity and enhancing nitrate adsorption and activation.^[^ ^93^ ^]^ Despite these advances, nonmetallic catalysts face challenges due to their intrinsically low activity and slow reaction kinetics, which currently limit their widespread application in NO_3_^−^RR. Nevertheless, they remain promising due to their economic and environmental advantages, especially in large‐scale, sustainable catalytic processes.

As illustrated by the examples above, introducing defects into nanoparticles is beneficial for the electrocatalytic reduction of nitrate. Defect engineering primarily involves disrupting or breaking the periodicity of the crystal structure, thereby altering the physicochemical properties of the catalyst. The electrocatalytic performance is closely related to both the type and quantity of defects. Similar to strain engineering, precisely controlling the nature and density of defects remains a significant challenge. Moreover, defect‐rich nanomaterials often exhibit high activity, which can compromise their stability—active sites may become deactivated over time. Therefore, achieving a balance between activity and stability is a key research focus in the field of defect engineering.

### 3.5 Compositional Engineering

#### 3.5.1 Alloying Engineering

To reduce the cost of catalysts and achieve nitrate reduction at low overpotentials for commercial‐scale denitrification, a critical challenge lies in developing electrocatalysts that are both cost‐effective and possess high activity, selectivity, and stability. Alloying noble metals with more affordable base metals offers a promising strategy. However, determining the optimal alloy composition is essential, as it directly influences catalyst performance, cost, and selectivity.

Among alloy materials, CuNi alloys have garnered significant attention due to their excellent corrosion resistance and catalytic performance. In particular, copper‐rich CuNi alloys, such as Cu_80_Ni_20_ and Cu_90_Ni_10_ prepared via electrodeposition, outperform their monometallic counterparts in alkaline electrochemical nitrate reduction, achieving a Faradaic efficiency up to 85.0%.^[^ ^96^ ^]^ This enhanced performance arises from the synergistic effect where nitrate preferentially adsorbs onto Cu sites, while Ni sites facilitate the adsorption of hydrogen atoms. The adsorbed H and NO_3_⁻ species subsequently react to form NO_2_⁻ and H_2_O, thereby promoting nitrate reduction and suppressing competitive hydrogen evolution.

**Figure 9:**
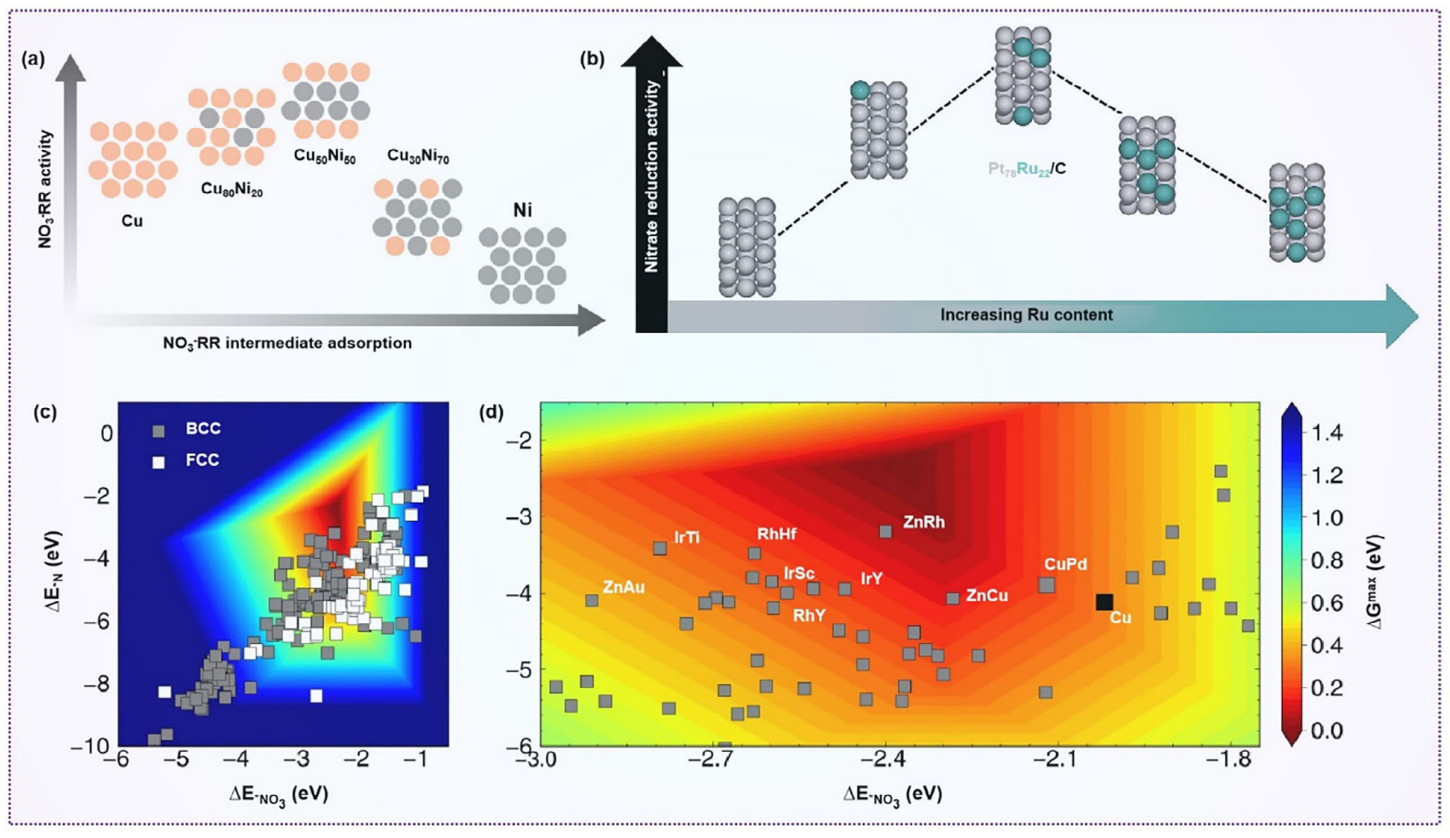
Alloying engineering on nitrate reduction. a) Relationship between intermediate adsorption and activity for Cu*_x_* Ni*_x_* in NO_3_^−^RR. Reproduced with permission.^[^ ^94^ ^]^ Copyright 2020, American Chemical Society. b) Relationship between Ru content and NO_3_^−^RR activity for Pt*_x_* Ru*_x_*/C. Reproduced with permission.^[^ ^95^ ^]^ Copyright 2020, Elsevier. c,d) DFT‐calculated adsorption energies of *NO_3_ and *N on (100)‐terminated B2 and (100)‐terminated FCC intermetallics. Reproduced with permission.^[^ ^58^ ^]^ Copyright 2022, The Author(s), Springer Nature.

Sargent et al. further investigated the structure–activity relationship of a Cu_50_Ni_50_ alloy, revealing that Ni alloying causes an upward shift of the Cu *d*‐band center toward the Fermi level. This shift alters the potential‐determining step from NO_3_⁻ adsorption to *NH_2_ hydrogenation, enhancing the adsorption energy of intermediates and reducing the overpotential (**Figure** **9**a).^[^ ^94^ ^]^ However, not all Cu‐based alloys are effective in ammonia production. For example, Xu et al. synthesized a CuPd alloy supported on a carbon dendritic framework that selectively converted nitrate to nitrogen with nearly 100% selectivity, thus prioritizing N₂ formation over ammonia.^[^ ^97^ ^]^

In addition to experimental studies, theoretical modeling has proven invaluable. Liu et al. used DFT to identify a simple thermodynamic descriptor, the binding energies of atomic O and N, to predict catalyst activity and selectivity for NO_3_^−^RR. Based on theoretical volcano plots, Pt_3_Ru emerged as one of the most promising alloy catalysts, outperforming even monometallic Pt.^[^ ^49^ ^]^ To validate these predictions, they synthesized a series of Pt*_x_* Ru*_y_*/C catalysts with varying Ru content (*x* = 48%–100%). All alloyed catalysts demonstrated better NO₃RR performance than Pt/C, with Pt_78_Ru_22_/C delivering the highest nitrate reduction activity (Figure 9b). These results suggest that alloying can be guided by the effect of composition on the binding energies of key intermediates, enabling more rational catalyst design.^[^ ^95^ ^]^

Interestingly, liquid metal catalysts, such as Galinstan (a room‐temperature liquid alloy of Ga, In, and Sn), have also emerged as highly active and selective catalysts for nitrate reduction. Galinstan achieved an NH₃ production rate of 2335 µg h^−1^ cm^−2^ with 100% Faradaic efficiency. Both computational and experimental analyses indicate that this exceptional activity is attributed to InSn alloy enrichment during the electrocatalytic process, which generates In₃Sn active sites. These sites not only favor nitrate reduction but also effectively suppress the competitive HER.^[^ ^98^ ^]^

Compared with random alloys, intermetallic nanocrystals offer unique advantages due to their well‐defined compositions and ordered structures, endowing them with distinct electronic properties and superior chemical stability (Figure 9c,d). Single‐crystal studies have confirmed that (100)‐oriented surfaces are more catalytically active than (111) facets for Cu‐based systems in nitrate‐to‐ammonia conversion. Building on these findings, researchers have synthesized (100)‐oriented CuPd nanocubes, where Pd incorporation shifts the Cu *d*‐band center upward, thereby enhancing NO_3_ bridge‐bidentate adsorption. Additionally, the hollow *N intermediate becomes destabilized due to Pauli repulsion from Pd subsurface *d*‐orbitals, facilitating efficient nitrogen species conversion to ammonia.^[^ ^58^ ^]^

Compared to monometallic catalysts, alloy materials offer several advantages for the electrocatalytic nitrate reduction reaction. In addition to reducing the use of noble metals, alloys can provide bifunctional or multifunctional active sites that facilitate complex multielectron reactions. However, interactions between different atoms in an alloy may influence the selectivity during the reaction. Given that nitrate reduction is a multistep process with diverse products, achieving high selectivity for specific pathways is particularly important. This can be regulated by tuning the composition and ratio of metals in the alloy. Moreover, adjusting the metal ratio in alloys may also impact the extent of side reactions, which should be considered. Additionally, alloy materials are prone to issues, such as lattice defects, dissolution, and corrosion of active sites during the reaction. These challenges are commonly encountered in nitrate electroreduction and must be addressed in future research.

#### 3.5.2 Hybrid Structure Engineering

Single‐metal catalysts often fail to meet the demands for high activity, selectivity, and stability in NO_3_^−^RR due to limited reaction pathway control. As nitrate reduction is a complex multistep, multielectron process, the design of multimetallic or hybrid catalysts can significantly enhance intrinsic catalytic performance through synergistic effects. Alloying and compositing enable modulation of the electronic structure, improve charge transfer, and optimize intermediate adsorption. In particular, bimetallic or doped systems provide active sites with tailored *d*‐band centers and reaction energetics.

**Figure 10:**
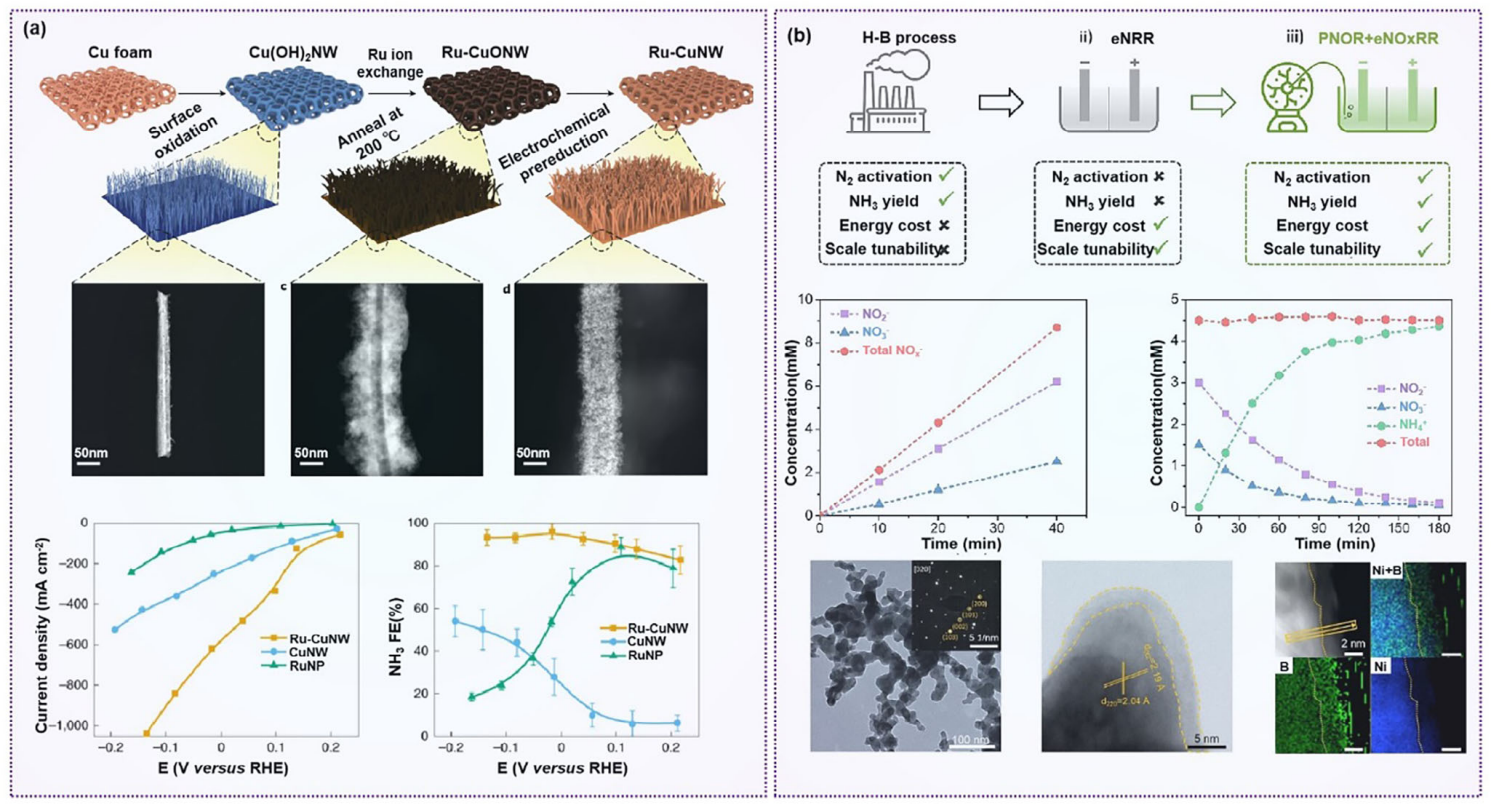
Hybrid structure engineering on nitrate reduction. a) The NO_3_^−^RR properties composition of Ru‐dispersed Cu nanowires (Ru‐CuNW), Cu nanowires (CuNW), and Ru nanoparticle (RuNP). Reproduced with permission.^[^ ^99^ ^]^ Copyright 2022, The Author(s), under exclusive licence to Springer Nature Limited. b) The NO*_x_* ^−^ yield as a function of plasma treatment time, the successive consumption of NO*_x_* ^−^, the ammonia production for electrochemical NO*_x_* ^−^ reduction reaction (eNO*_x_* RR) of Ni_3_B@NiB_2.74_ catalyst. Reproduced with permission.^[^ ^100^ ^]^ Copyright 2021, Wiley‐VCH.

For instance, monometallic Cu favors nitrate reduction to nitrite, while Pt nanoparticles drive the subsequent conversion of nitrite to ammonia. Building on this, Cerron‐Calle et al. designed 3D Cu‐Pt bimetallic electrodes, achieving a nitrate conversion efficiency of ≈94%, compared to 55% for Cu‐only foams.^[^ ^101^ ^]^ Similarly, Fe doping into Cu (e.g., Cu_49_Fe_1_) deepened the Cu 3*d* energy level, optimizing intermediate adsorption and promoting nitrate reduction in neutral media.^[^ ^102^ ^]^ The coexistence of discrete Pd and Cu clusters also affects Faradaic efficiency, where Cu(111) serves as the primary nitrate reduction site, and Pd(111) facilitates hydrogen atom generation via water dissociation, supplying hydrogen for NH₃ synthesis.^[^ ^103^ ^]^ To tackle real‐world conditions such as low nitrate concentrations in industrial or drinking water, Chen et al. developed Ru‐dispersed Cu nanowires, achieving over 99% nitrate conversion and >90% Faradaic efficiency in solutions as dilute as 50 ppm NO_3_⁻ (**Figure** **10**a).^[^ ^99^ ^]^

Copper oxides, particularly CuO and Cu_2_O, have garnered significant attention in the field of nitrate electroreduction due to their favorable redox properties and catalytic versatility. Wang et al. synthesized CuO nanowires and investigated their catalytic behavior, revealing that CuO undergoes a phase transformation into Cu/Cu_2_O, which constitutes the true active phase. This dynamic transformation facilitates interfacial electronic transfer from Cu_2_O to Cu, promoting the generation of reactive intermediates while concurrently suppressing the competitive HER. Consequently, both the Faradaic efficiency and ammonia selectivity were significantly enhanced.^[^ ^104^ ^]^ Ren et al. designed Cu@Cu_2+1_O core–sheath nanowires, which demonstrated superior catalytic performance for nitrate‐to‐ammonia conversion. In this architecture, the inner metallic Cu core supports efficient electron transfer, while the outer oxide sheath, rich in catalytically active sites, facilitates intermediate adsorption and transformation. The interfacial interaction between the metallic core and oxide shell effectively modulates the Cu *d*‐band center, optimizing adsorption energies for key intermediates and improving overall reaction kinetics. Compared to Cu@Cu_2+1_O nanoparticles, this nanowire configuration boosted the ammonia production rate from 244.5 to 576.53 µg h^−1^ mg^−1^ and increased the FE from 27.9% to 87.07%.^[^ ^105^ ^]^ Fu et al. demonstrated that modulating the Cu₂O/Cu interface can significantly influence nitrite adsorption behavior. Their study revealed that tuning this interface weakens the binding energy of adsorbed nitrite, facilitating its surface diffusion. DFT calculations further indicated that the Cu_2_O/Cu interface upshifts the Cu *d*‐band center, enhancing electron transfer to nitrate and thereby improving the reduction kinetics.^[^ ^106^ ^]^

Furthermore, active site regulation in metal oxides also offers a promising approach to improve the Faradaic efficiency, selectivity, and ammonia yield in nitrate reduction. For example, Qiu et al. incorporated CuO*_x_* species into TiO_2_ nanotubes (TiO_2_ NTs/CuO*_x_*), effectively trapping NO₂⁻ intermediates within the nanotube structure. This confinement effect suppresses intermediate diffusion and promotes complete conversion of NO_3_⁻ to NH_3_ with improved yield and Faradaic efficiency.^[^ ^107^ ^]^ Cai et al. designed a dual‐site Ru&Cu/Cu_2_O catalyst to enhance nitrate adsorption and drive its preferential reduction to ammonia.^[^ ^108^ ^]^

However, while Cu‐based catalysts often suffer from high overpotentials in practical applications, Ni‐based catalysts, despite their strong intermediate adsorption capability, also exhibit considerable hydrogen evolution activity, which limits their overall selectivity and ammonia yield. Li et al. synthesized a boron‐rich core–shell nickel boride catalyst (Ni_3_B@NiB_2.74_) for two‐step ammonia production (Figure 10b). The high surface boron content facilitates nitrate adsorption, improves catalytic activity and selectivity, inhibits the competing HER, and protects surface Ni from oxidation.^[^ ^100^ ^]^ This demonstrates the potential of surface engineering in tuning the reaction pathway and enhancing catalyst robustness. In pursuit of scalable and efficient nitrate reduction, Zheng et al. developed a Ni(OH)_2_@Ni electrode through in situ activation of pristine nickel. This hybrid electrode exhibited high catalytic activity, selectivity, and operational stability at both laboratory and pilot scales.^[^ ^109^ ^]^

Besides, Fe_3_O_4_, a low‐cost, chemically stable, and conductive iron oxide, has also been utilized for efficient nitrate reduction. Fe_3_O_4_ particles grown in situ on stainless steel substrates demonstrated excellent performance, achieving a Faradaic efficiency of 91.5% and an ammonia yield of 10145 µg h^−1^ cm^−2^, highlighting the potential of iron‐based systems in practical applications.^[^ ^110^ ^]^

The activity and selectivity of hybrid electrocatalysts are largely determined by their multicomponent composition, which includes materials of varying types and chemical compositions. In supported catalyst systems, beyond the individual contributions of each component to the adsorption and desorption of intermediates, the interaction between the support and the active species can significantly influence charge transfer, geometric configuration, interfacial strain, spillover effects, and synergistic interactions. Collectively, these factors play a critical role in shaping the overall electrocatalytic performance for nitrate reduction. Nevertheless, distinguishing the precise function of each component remains a major challenge, as the interactions within composite catalysts are complex, intertwined, and often occur simultaneously. Therefore, fine‐tuning these interactions at the atomic level is crucial for the rational design and development of high‐performance electrocatalysts for nitrate reduction.

#### 3.5.3 Doping Engineering

Doping regulation is a powerful strategy in electrocatalysis for tuning the electronic and structural properties of catalysts. Atomic‐level doping can modulate the electronic structure, Gibbs free energy (Δ*G*_H_*), and *d*‐band center of the active site, significantly improving reaction kinetics. Moreover, doping influences vacancy concentration, phase transitions, surface wettability, electronic band structure, charge distribution, conductivity, adsorption strength, and the configuration of adsorbed species.^[^ ^113^, ^114^, ^115^ ^]^ Since the adsorption and desorption behaviors of intermediates, particularly *NO and *NOH, are rate‐determining steps in NO_3_^−^RR, these surface properties are crucial to achieving high catalytic performance.

**Figure 11:**
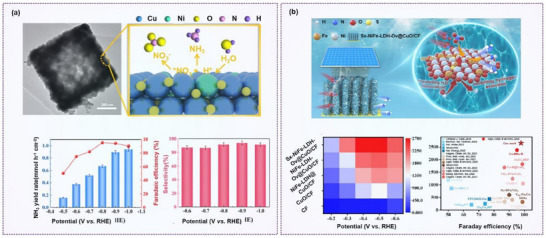
Doping engineering on nitrate reduction. a) Ammonia production rate and Faradaic efficiency of hollow square morphology (Ni–CuO) NO_3_^−^RR performance at different potentials. Reproduced with permission.^[^ ^111^ ^]^ Copyright 2025, American Chemical Society. b) Schematic illustration of nitrate reduction using S*_x_*‐NiFe‐LDH‐O*_v_* @CuO/CF and NO_3_^−^RR performance. Reproduced with permission.^[^ ^112^ ^]^ Copyright 2024, Elsevier.

Combining metals with complementary electronic properties can create synergistic effects. For instance, Li et al. synthesized a hollow square‐shaped Ni‐doped copper oxide catalyst (Ni*─*CuO), which demonstrated a high NH_3_ yield of 0.94 mmol h^−1^ cm^−2^ and a Faradaic efficiency of 95.26% for NO_3_^−^RR (**Figure** **11**a).^[^ ^111^ ^]^ This performance is attributed to the enhanced nitrate adsorption enabled by the Ni*─*Cu interface and the structural advantages of the hollow morphology.

As nitrate is a weak Lewis base, catalysts featuring abundant Lewis acid sites can facilitate its adsorption. Boron, with its empty 2*p* orbital, serves as an effective Lewis acid dopant.ingly, boron‐doped electrocatalysts have shown strong promise for selective nitrate reduction. BCD/NiCo_2_O_4_/CC catalysts, created by introducing boron‐doped carbon dots (BCD), significantly enhanced nitrate adsorption and suppressed the competing HER, achieving high selectivity and Faradaic efficiency.^[^ ^116^ ^]^ Boron‐doped diamond (BDD) materials have also attracted attention due to their exceptional stability, corrosion resistance, and wide electrochemical window. Despite their high Faradaic efficiency, BDD tends to favor nitrogen generation over ammonia. Strategies such as adjusting the B doping ratio, sp^3^/sp^2^ carbon content, support conductivity, and grain size have been employed to improve both NH₃ selectivity and HER suppression.^[^ ^117^, ^118^, ^119^, ^120^, ^121^ ^]^

In another study, Li et al. synthesized fluorine‐doped carbon (FC) by calcining discarded cigarette filters in F‐containing solutions. The resulting material exhibited a Faradaic efficiency of 20% and an ammonia production rate of 23.8 mmol h^−1^ mg_cat_^−1^, which were twofold and fourfold higher, respectively, than those of undoped carbon.^[^ ^122^ ^]^ Further, boron‐doped vertically aligned NiCo₂O₄ pillars decorated with carbon dots (BCDs) achieved 100% Faradaic efficiency for nitrate reduction. This remarkable performance is ascribed to boron‐induced enhancement of nitrate adsorption and the suppression of HER, thereby greatly improving NH_3_ selectivity.^[^ ^116^ ^]^ Doping can also introduce beneficial surface defects, which facilitate H₂O dissociation and H* generation, addressing proton deficiency during neutral‐condition NO₃⁻ reduction.

Ge et al. introduced sulfur doping to induce lattice distortion in NiFe‐layered double hydroxides (NiFe‐LDH), thereby promoting defect formation. The resulting S*x*‐NiFe‐LDH‐O*_v_* @CuO/CF catalyst showed a Faradaic efficiency of 97.8% when treating 100 mg N L⁻¹ nitrate wastewater (Figure 11b).^[^ ^112^ ^]^ Sulfur‐mediated defects were found to shift the *d*‐band center of Ni and Fe sites, promoting nitrate adsorption while suppressing H* binding. This optimization enhances *NO hydrogenation and boosts catalytic activity even under low nitrate concentrations.

Doping engineering has greatly expanded the application potential of nanostructured electrocatalysts and is equally applicable to nitrate electroreduction. However, synthesizing heteroatom‐doped catalysts with uniformly distributed dopants and precise compositions remains a significant challenge. These challenges primarily lie in controlling the type, location, and quantity of the dopants. It also remains unclear whether the doped atoms act merely as promoters or serve as actual active sites. Moreover, factors such as the distance between adjacent dopant atoms and the changes in chemical bonding around the doped sites caused by the introduction of dopants, as well as the inherent properties of the catalysts and their structural evolution during the reaction process, are all critical issues that warrant further investigation.

### 3.6 Confinement Engineering

Confinement engineering refers to the deliberate design and spatial restriction of active sites within confined environments, such as porous structures, nanocages, layered frameworks, or defect‐rich matrices, to regulate electronic transfer capabilities, atomic arrangement, molecular structure, coordination environment, dielectric constant, and mass transport during catalysis. This concept goes beyond the construction of single‐atom catalysts (SACs) and can include single‐atom confinement, encapsulation, or embedding of nanomaterials, and hierarchical confinement. SACs have been extensively studied in the field of nitrate reduction due to their advantages over bulk catalysts, including maximized atomic utilization, well‐defined coordination environments, and enhanced activity and selectivity. Their isolated active sites allow for precise control of reaction pathways, making them ideal candidates for the selective reduction of nitrate to ammonia.

**Figure 12:**
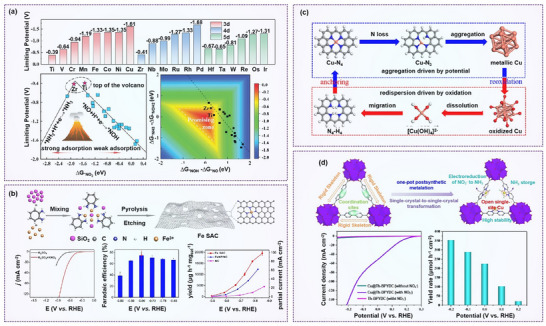
Confinement engineering on nitrate reduction. a) Summary of limiting potentials on TM/*g*‐CN for NO_3_^−^RR, and volcano plot. Reproduced with permission.^[^ ^123^ ^]^ Copyright 2020, Wiley‐VCH. b) Schematic illustration of the synthesis of Fe SAC and NO_3_^−^RR performance. Reproduced with permission.^[^ ^124^ ^]^ Copyright 2021, The Author(s), Springer Nature. c) Diagrammatic representation of the proposed mechanisms: potential‐induced aggregation and redispersion under oxidative conditions. Reproduced with permission.^[^ ^125^ ^]^ Copyright 2022, American Chemical Society. d) Schematic illustration of the synthesis of metal–organic frameworks by anchoring single Cu atoms on a single‐crystal Th‐based MOF (Cu@Th‐BPYDC) and NO_3_^−^RR performance. Reproduced with permission.^[^ ^126^ ^]^ Copyright 2021, American Chemical Society.

Niu et al. proposed the feasibility of graphitic carbon nitride (*g*‐CN)‐supported SACs for NO_3_^−^RR through DFT calculations (**Figure** **12**a). Among a range of transition metals, Ti/*g*‐CN and Zr/*g*‐CN exhibited the most favorable performance, sitting near the apex of the NO_3_RR volcano plot when using ΔG_*NO3_ as a descriptor. These catalysts demonstrated low limiting potentials and high selectivity toward NH₃ formation with significant suppression of undesired by‐products.^[^ ^123^ ^]^

Zero‐valent iron is traditionally used in nitrate remediation; however, it suffers from surface oxidation and low conductivity. In contrast, atomic Fe sites, particularly when coordinated with nitrogen species, form stable Fe*─*N*x* configurations that not only resist oxidation but also create specific nitrate‐preoccupied transition centers, reducing competitive H₂O adsorption.^[^ ^127^ ^]^ This strategy effectively enhances selectivity and activity in aqueous nitrate reduction. Wu et al. synthesized a highly efficient Fe single‐atom catalyst (Fe SAC), which isolated the Fe active centers and prevented N*─*N coupling, a common pathway that leads to nitrogen formation in multiatom systems. This structural configuration enabled a high NH₃ yield of 20 000 µg h^−1^ cm_cat^−2^ and a Faradaic efficiency of ≈75% (Figure 12b).^[^ ^124^ ^]^

While metallic Cu is a widely recognized electrocatalyst for NO_3_^−^RR, it suffers from low long‐term stability, low activity, and nitrate accumulation. To address these limitations, Zhu et al. developed a Cu‐N‐C‐800 catalyst, where single Cu atoms were embedded in nitrogen‐doped carbon frameworks. Experimental results and DFT calculations revealed that the Cu*─*N coordination not only enhanced the adsorption of NO_3_⁻ and NO_2_⁻ but also inhibited nitrite release, thereby promoting complete reduction to NH_3_ and N_2_.^[^ ^128^ ^]^

However, one critical yet often overlooked aspect of SACs is the dynamic restructuring of active sites under electrochemical conditions. Using operando X‐ray absorption spectroscopy and advanced electron microscopy, Yang et al. demonstrated that Cu*─*N_4_ sites gradually transform into Cu*─*N_3_, near‐free Cu^0^ single atoms, and eventually Cu^0^ nanoparticles (≈5 nm) as the applied potential shifts. These Cu nanoparticles were identified as the true active sites for NO_3_⁻RR. Importantly, the restructuring was reversible. After the reaction, the nanoparticles disassembled and returned to their original atomic configuration, masking the dynamic structural evolution during catalysis (Figure 12c).^[^ ^125^ ^]^ This discovery is pivotal in advancing our understanding of SAC behavior and the nature of active sites under reaction conditions.

Gao et al. extended SAC design to metal–organic frameworks (MOFs) by anchoring single Cu atoms on a single‐crystal Th‐based MOF (Th‐BPYDC). The resulting Cu@Th‐BPYDC catalyst exhibited an open, unsaturated Cu coordination environment that not only facilitated NO_3_⁻ to NH_3_ conversion but also acted as a Lewis acid site for NH₃ adsorption and storage. This dual‐functionality highlights the versatility of single‐site SACs. However, a major limitation is the time‐consuming synthesis, in which up to 7 days is required, hindering practical scalability despite the high Faradaic efficiency (Figure 12d).^[^ ^126^ ^]^

This section on confinement engineering primarily focuses on the influence of single‐atom catalysts on nitrate reduction. Both theoretical calculations and experimental studies have demonstrated that single‐atom catalysts can enhance the efficiency of nitrate reduction. However, precisely regulating and monitoring confinement at the molecular and atomic levels remains challenging. At these scales, confinement engineering can be manipulated by tuning the coordination environment and altering charge transfer capabilities, thereby controlling the size and catalytic performance. Such precise control can optimize the intrinsic electrical conductivity and catalytic activity of the electrocatalyst. Additionally, the confined space can influence mass transport during the catalytic process. Interfaces formed by the confined supports can also serve as critical active sites for the catalytic reaction. Moreover, the self‐reconstruction of single‐atom catalysts during the reaction process cannot be overlooked. Potential‐induced restructuring of active sites plays a critical role in determining the catalytic mechanism of single‐atom catalysts. In situ characterization techniques, by enabling real‐time tracking of the dynamic evolution of catalysts under operating conditions, are essential for identifying the true active sites and gaining mechanistic insights into the electrocatalytic nitrate reduction reaction. Such understanding is pivotal for the rational design and optimization of more stable and efficient single‐atom catalysts systems.

### 3.7 Biomimetic Engineering

Traditionally, nitrate removal from water has been achieved through physicochemical techniques or microbial degradation. However, microbial processes are inherently slow and difficult to maintain over extended periods due to the sensitivity of microorganisms to environmental fluctuations. In a pioneering study, Mellor et al. introduced a bioelectrochemical approach for efficient nitrate removal, utilizing the catalytic reduction capabilities of immobilized enzymes.^[^ ^131^ ^]^ Their system consisted of an electrobioreactor employing a two‐stage process: immobilizing enzymes while preserving their activity, and effectively transferring reducing power to these immobilized enzymes. The matrix contained coimmobilized NADH: nitrate reductase (from corn), crude nitrite reductase, and N_2_O reductase (from Rhodopseudomonas), along with electron carrier dyes. These were embedded within a polymer matrix adhered to the cathode surface. When a low voltage was applied, nitrate‐containing water flowed through this active layer, allowing stepwise enzymatic reduction of nitrate to dinitrogen (N_2_) via nitrite (NO_2_⁻). In nature, nitrate reduction to ammonia is a multistep enzymatic process, where nitrate reductase first reduces NO_3_⁻ to NO_2_⁻, followed by further reduction by nitrite reductase or nitrogenase to produce NH_3_.

**Figure 13:**
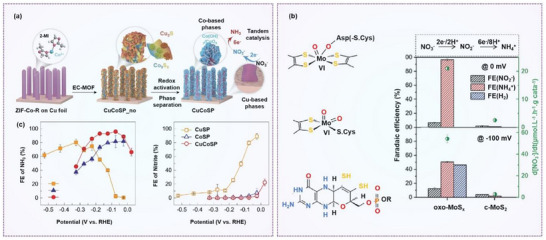
Biomimetic engineering on nitrate reduction. a) Biomimetic microbial‐inspired tandem conversion of NO_3_^−^ to NH_3_: Schematic illustration of the synthesis of Cu/Co‐based binary tandem catalyst and NO_3_^−^RR performance. Reproduced with permission.^[^ ^129^ ^]^ Copyright 2022, The Author(s), Springer Nature. b) Biomimicry of enzyme structures: Schematic diagram of active‐site structures and nitrate reduction by oxo‐MoS*_x_* and c‐MoS_2_. Reproduced with permission.^[^ ^130^ ^]^ Copyright 2020, Wiley‐VCH.

Inspired by this biological cascade, He et al. developed a tandem electrocatalyst that mimics the enzyme‐like sequential reduction mechanism. By electrochemically transforming pre‐synthesized Cu*─*Co binary metal sulfides on copper foil, they formed a core–shell structure of Cu/CuO*_x_* and Co/CoO. In this system, Cu‐based domains preferentially catalyze the reduction of NO_3_⁻ to NO_2_⁻, while Co‐based domains facilitate the subsequent conversion of NO_2_⁻ to NH_3_. This tandem configuration effectively decouples the reaction pathway and significantly enhances both the kinetics and selectivity of nitrate reduction. The catalyst achieved a Faradaic efficiency of 93.3% ± 2.1% for NH₃ production at pH 13, with an NH_3_ yield rate of 1.17 mmol cm^−2^ h^−1^ at −0.175 V (**Figure** **13**a).^[^ ^129^ ^]^

Further biomimetic inspiration stems from the structural sophistication of natural nitrate reductases, which exhibit high catalytic efficiency due to their precise coordination environments. Building on this concept, Chen et al. synthesized a Cu‐incorporated organic molecular solid (3,4,9,10‐perylenetetracarboxylic dianhydride, O‐Cu*─*‐PTCDA), which displayed controlled proton/electron transfer dynamics, while suppressing the competing HER. The optimized molecular catalyst achieved a maximum Faradaic efficiency of 85.9% for ammonia production.^[^ ^132^ ^]^

In natural systems, all known nitrate reductases employ Mo (molybdenum) as their catalytic center. The active site typically comprises a mononuclear Mo coordinated by an oxo group, a dithiolene‐thiol ligand, and one or more molybdopterin cofactors, with additional coordination from sulfur or oxygen ligands on nearby amino acid residues. While Mo‐based catalysis is highly effective, it is traditionally limited to organic solvents due to low water stability. Addressing this limitation, Li et al. designed a Mo‐based inorganic catalyst composed of oxygen‐doped molybdenum sulfide, serving as a functional analogue to natural nitrate reductases (Figure 13b). Unlike many previous systems, this catalyst operates effectively under neutral aqueous conditions, preserving Mo‐based catalytic advantages while improving stability and scalability for practical application.^[^ ^130^ ^]^

Significant progress has been made in the design and synthesis of nitrate reductase mimics, inspired by microorganisms in nature that decompose nitrate. These mimics effectively replicate various characteristics of natural enzymes. Some electrocatalysts are designed based on the active sites of enzymes themselves, while others employ tandem catalysis inspired by the cascade reactions of natural enzymes, both of which enhance the electrocatalytic performance of nitrate reduction. Biomimetic chemistry, drawing inspiration from the structural and synthetic strategies found in nature, offers a highly efficient, environmentally friendly, and economically viable approach to designing electrocatalysts for nitrate reduction. However, current biomimetic catalysts typically replicate only one aspect of natural enzymes and still fall short of mimicking the full complexity of their structures. To bridge this gap, machine learning and computational modeling can be employed to construct catalysts that more closely resemble natural enzymes, enabling even more efficient nitrate reduction.

### 3.8 Other Engineering

In addition to the above‐mentioned engineering strategies that can enhance the electrocatalytic performance for NO_3_^−^RR, there are also other engineering approaches aimed at improving the ammonia yield, Faradaic efficiency, and selectivity of the nitrate reduction reaction. For example, to elucidate the role of surface‐modified oxygen in promoting nitrate‐to‐ammonia conversion, Wang et al. synthesized ultrathin cobalt oxide (CoO*_x_*) nanosheets rich in surface oxygen groups. These oxygen‐rich sites effectively stabilized the adsorption of hydrogen on the CoO*_x_* surface and suppressed the competing HER, thereby enhancing the Faradaic efficiency of the nitrate reduction reaction. Specifically, at −0.3 V, the ammonia yield reaches 82.4 ± 4.8 mg h^−1^ mg_cat_^−1^, with a Faradaic efficiency of up to 93.4 %± 3.8%.^[^ ^133^ ^]^ Another innovative approach involves the creation of built‐in electric fields at the interface of semiconductor materials. When a semiconductor is coupled with a secondary material, the charge transfer across the interface continues until Fermi level alignment is achieved, resulting in an internal electric field. Based on this principle, Sun et al. constructed a composite catalyst by integrating CuCl with TiO_2_ (CuCl_BEF), taking advantage of the strong chemical bonding between Cu and O. The built‐in electric field facilitated stronger nitrate adsorption on the catalyst surface and reduced the activation energy of the rate‐limiting step. This enabled efficient ammonia production from the low‐concentration nitrate solutions with high selectivity.^[^ ^134^ ^]^ The successful application of built‐in electric fields presents a promising strategy for improving nitrate reduction performance. Enrichment strategies have been proven effective in promoting electrochemical nitrate reduction. Song et al. investigated the enrichment effect on nitrate reduction using an electrocatalyst composed of copper nanoparticles encapsulated in a porous carbon framework (Cu@C). The Cu@C catalyst achieved a Faradaic efficiency of up to 72.0% for ammonia production from nitrate, which is 3.6 times higher than that of bare Cu nanoparticles. In nitrate solutions of varying concentrations, the Faradaic efficiency of Cu@C consistently outperformed that of the Cu catalyst.^[^ ^135^ ^]^ This enhancement is attributed to the enrichment effect of the porous carbon layer, which facilitates the accumulation of nitrate ions on the catalyst surface, thereby improving mass transfer and accelerating the nitrate reduction reaction. This enrichment strategy provides valuable insights for improving nitrate reduction performance, particularly under low‐concentration conditions.

## 4 Electrolyte Design for Efficient NO_3_^−^RR

**Figure 14:**
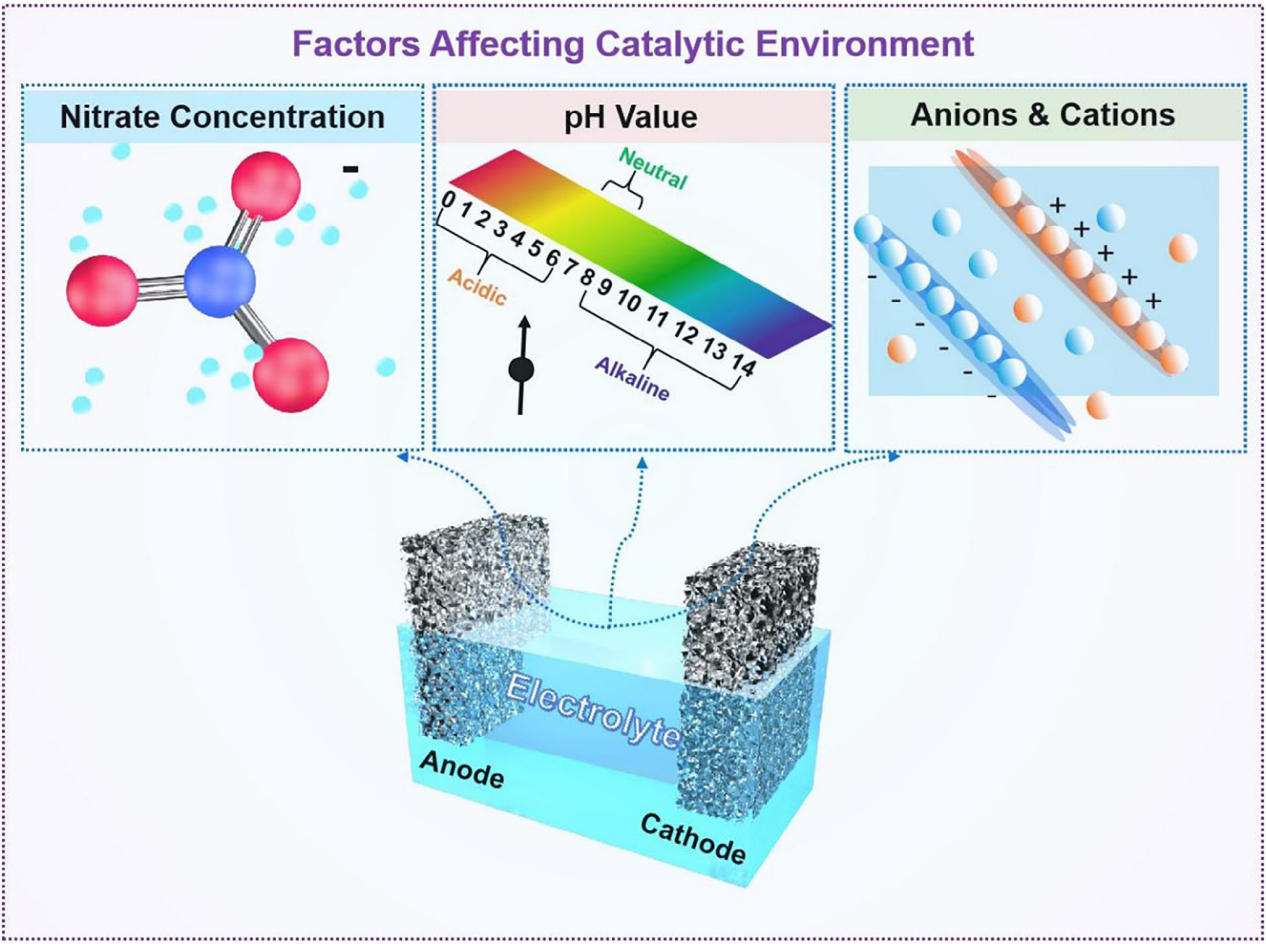
Summary on factors affecting catalytic environment during NO_3_^−^RR.

Although the working electrode potential defines the thermodynamic feasibility of the electrochemical process, and the intrinsic properties of the catalyst material primarily determine its catalytic performance, the reaction environment, particularly the supporting electrolyte, also plays a crucial role in modulating activity, selectivity, and stability. The electrolyte influences not only the ionic conductivity and pH of the system but also the availability of protons, the adsorption/desorption behavior of intermediates, and the electrochemical double‐layer structure, all of which collectively impact the overall efficiency of the catalytic reaction (**Figure** **14**).

### 4.1 Nitrate Concentration and pH

**Figure 15:**
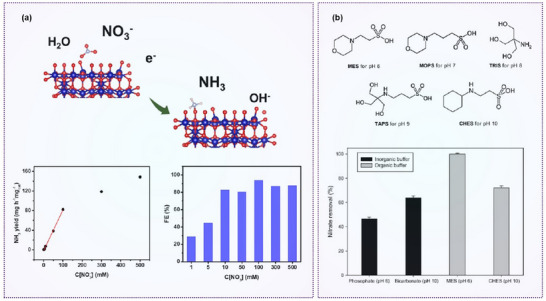
Effect of nitrate concentration and pH on nitrate reduction performance. a) Schematic diagram of structures and ammonia yield and FE of CoO*_x_* in electrolytes with different nitrate concentrations. Reproduced with permission.^[^ ^133^ ^]^ Copyright 2021, American Chemical Society. b) Chemical structures of different buffers and nitrate reduction in inorganic and organic buffers at pH 6 and 10. Reproduced with permission.^[^ ^142^ ^]^ Copyright 2013, Elsevier.

The electrochemical nitrate reduction mechanism is strongly influenced by the concentration of nitrate reactants and the acidity of the electrolyte. The indirect nitrate reduction autocatalytic pathway is observed primarily at high nitrate concentrations (>1 m) and in highly acidic media.^[^ ^49^, ^136^ ^]^ In contrast, most studies on nitrate reduction focus on reactions occurring at lower reactant concentrations (<1 m), which proceed via a direct reaction mechanism. These reactions can follow two primary pathways: electron transfer reduction and atomic hydrogen reduction. Given that nitrogen can adopt a wide range of electronic valence states, its intermediates and products are highly complex. However, thermodynamically, nitrate is ultimately converted to nitrogen and ammonia. The electrochemical reduction of nitrate generally begins with the adsorption of nitrate ions on the electrode surface, making the concentration of nitrate a critical factor. At low nitrate concentrations in the supporting electrolyte, the adsorption of competing species on the electrode surface becomes a key factor that can hinder nitrate reduction. Conversely, at high nitrate concentrations, the catalyst's activity becomes the dominant factor influencing the nitrate reduction process.^[^ ^137^ ^]^ Wang et al. investigated the effect of nitrate concentration using ultrathin CuO nanosheets.^[^ ^133^ ^]^ When the concentration was below 100 mm, the ammonia yield exhibited a linear relationship with the nitrate concentration, with a slope of 0.83, indicating that the nitrate reduction is a first‐order reaction with respect to nitrate concentration (**Figure** **15**a). When the nitrate concentration dropped below 10 mm, the Faradaic efficiency decreased sharply due to the competing HER. In contrast, when the nitrate concentration was significantly increased, the contribution of HER was likely reduced, and the Faradaic efficiency could be maintained at relatively high values. Based on these results, regulating the nitrate concentration can also help achieve maximum efficiency in nitrate reduction.

In the electrocatalytic nitrate reduction reaction, along with the HER, proton consumption or hydroxide production occurs, which can alter the pH of the electrolyte. This change in pH is influenced by reaction rates, electrolyte composition, and mass transport properties. As a result, the pH of the electrode environment can vary significantly, thereby affecting the selectivity and activity of nitrate reduction.^[^ ^138^, ^139^ ^]^

Nitrate reduction leads to the formation of ammonia (NH_4_⁺), with its behaviors highly influenced by the pH of the solution. In acidic solutions, nitrate is completely reduced to NH_4_⁺, while in alkaline solutions, it forms soluble NH_4_OH. The p*K*a of NH₄OH is 9.27, which means that at pH values above 9.27, NH_4_OH will predominate in the solution. However, NH_3_ spillover can occur in these conditions, resulting in a discrepancy between the measured ammonia concentration and the actual ammonia produced. This is because ammonia (NH_3_) can escape into the gas phase, especially at higher pH, leading to underreporting of the total ammonia concentration.

To accurately measure ammonia production, the excess NH_3_ is often recovered and converted back into NH_4_⁺ by lowering the pH, thereby enabling proper quantification. This adjustment allows for more accurate detection of ammonia, accounting for the error induced by solution pH.^[^ ^52^, ^140^ ^]^

The pH of the solution significantly affects the reaction pathway and product distribution. In the reduction of nitrate to ammonia, one potential by‐product is hydroxylamine (NH_2_OH). The ratio of ammonia to hydroxylamine is influenced by the rate at which hydroxylamine is reduced.^[^ ^141^ ^]^ Through the reduction of nitrate by Cu(111) and Cu(100), Pérez‐Gallent, et al. found that when using Cu(111) and Cu(100) catalysts, only Cu(100) in alkaline medium produces hydroxylamine, while both Cu(111) and Cu(100) in acidic media yield ammonia. The slower reduction rate under alkaline conditions contributes to this difference in product distribution.^[^ ^78^ ^]^

In addition to the pH, other factors such as applied potential and nitrate concentration also affect the selectivity of the nitrate reduction reaction. McEnaney et al. found that for achieving high selectivity in nitrate reduction at a Ti cathode, high concentrations of both nitrate ions and protons (H⁺) are necessary. This highlights the importance of controlling these parameters for optimizing the reaction and improving the selectivity toward ammonia production.^[^ ^55^ ^]^

In solutions with different pH values, the surface of the catalyst becomes covered by different adsorbed species. At low pH values, the catalyst surface is primarily covered by hydrogen ions, whereas at high pH values, hydroxides, and potentially oxides are adsorbed. These adsorbed species compete with the formation of nitrate adsorption, which is one of the reasons why nitrate reduction performance decreases rapidly in solutions with very low pH.

For Pd‐Cu catalysts, the maximum electrocatalytic activity is observed around a solution pH of 9. As the catalytic reaction progresses, the rising concentration of OH⁻ leads to an increase in the solution's pH. The increase in pH leads to the formation of hydroxides, which can competitively bind to the active sites of the bimetallic catalyst, preventing the adsorption of nitrate and nitrite and thereby restricting nitrate reduction activity. Additionally, strongly adsorbed oxidizing species can block the active sites on the catalyst surface, and metal sites may even become oxidized.^[^ ^143^, ^144^ ^]^

This issue is particularly prominent with Pd catalysts, where the surface becomes progressively covered by strongly adsorbed oxidizing species as the pH increases. This prevents nitrogen species from diffusing to the catalyst surface, resulting in fewer available palladium active sites. To overcome this challenge, Pintaret et al. suggested a solution involving continuous denitration in separate single‐flow fixed‐bed reactor units functioning in a batch‐recycle mode. In the initial reactor, a Pd‐Cu bimetallic catalyst reduces nitrate ions to nitrite at pH 12.5, achieving 93% selectivity. The second reactor, operating at low pH values (3.7 and 4.5), enables the hydrogenation of nitrite to nitrogen over a Pd monometallic catalyst.^[^ ^145^ ^]^

The reduction of nitrate by Pt occurs only at low pH, specifically below pH 4. In contrast, the reduction of nitrate by Rh occurs over a much broader pH range. For Pt, HNO_3_ is considered the only reducible species, while both HNO_3_ and nitrate anions are reducible on Rh. Rh has higher catalytic activity for nitrate reduction than Pt because, in the absence of protons in solution, Rh can activate nitrate directly.^[^ ^50^, ^146^ ^]^

Titanium (Ti) is known for its strong corrosion resistance in acidic, alkaline, and high salinity solutions. It has poor hydrogen evolution performance, requiring a higher hydrogen evolution potential compared to many metals. This makes Ti suitable for use in various saline solutions, where it can be explored for different electrochemical reduction reactions under a wide potential window.^[^ ^147^, ^148^ ^]^

McEnaney et al. used a matrix to comprehensively evaluate the effects of solution pH, nitrate concentration, and applied potential on nitrate electroreduction, specifically focusing on the electroreduction of ammonia by nitrate on titanium electrodes. Their results showed that current density under extreme pH conditions (both very low and very high) was significantly higher than at moderate pH levels. They observed that an increase in proton concentration generally corresponds to higher Faradaic efficiency of ammonia production. This implies that Faradaic efficiency is typically higher in acidic solutions compared to alkaline solutions.^[^ ^55^ ^]^

Similarly, Dortsiou et al. investigated the effect of six metal electrodes (Sn, Bi, Pb, Al, Zn, In) on nitrate reduction at different pH values. They found that in the pH range of 0–4, the nitrate reduction rate increased linearly with the concentration of hydronium ions (H_3_O⁺) in the solution. However, this relationship was independent of pH at higher pH values. They also observed that pH influences the formation of the final product species: in the range of pH 0–4, ammonia and hydroxylamine were mainly produced, while at pH >4, the main products included nitrogen, nitrous oxide, ammonia, and nitrite.^[^ ^149^ ^]^

Wang et al. discovered that Pd/Sn‐modified activated carbon fiber electrodes at elevated pH values showed a greater suppression of nitrite reduction compared to nitrate, while having little impact on the final ammonia formation.^[^ ^150^ ^]^ This suggests that pH primarily affects the nitrate reduction pathway, particularly the competition between nitrate and nitrite reduction.

Buffer solutions with high concentrations of buffer ions are commonly employed to mitigate pH changes at the interface and in the solution during electrochemical reactions. To control pH, both inorganic and organic buffers are widely used. For inorganic buffer solutions, compounds such as carbon dioxide, formic acid, and hydrochloric acid are commonly utilized to maintain a stable pH of the solution.^[^ ^151^, ^152^, ^153^, ^154^ ^]^ Phosphate buffer solution, when used as the electrolyte for NO_3_^−^RR, not only serves as a supporting electrolyte to supply protons and maintain a stable pH level, but also facilitates proton transfer at the electrocatalyst–electrolyte interface. This, in turn, promotes the hydrogenation of nitrate reduction intermediates and enhances the efficiency of ammonia production.^[^ ^155^ ^]^ SungjunBae et al. compared the electrocatalytic performance of nitrate reduction in organic and inorganic buffer systems at the same pH (Figure 15b).^[^ ^142^ ^]^ The study found that organic buffer systems, 2‐(*N*‐morpholino)ethanesulfonic acid (for pH 6) and *N‐*Cyclohexyl‐2‐aminoethanesulfonic acid (for pH 10), showed better nitrate removal performance than inorganic buffer systems, such as phosphate (for pH 6) and bicarbonate (for pH 10). However, the study also observed that nitrate removal decreased significantly when using Trizma buffer at pH 8. This reduction in performance was attributed to copper etching on the catalyst surface, which negatively impacted the reaction efficiency.

Most NO_3_^−^RR studies have been conducted under laboratory conditions, typically using fixed nitrate concentrations and specific pH values. Although a variety of simulated wastewater types have been tested, there are still many practical challenges when dealing with real wastewater. High concentrations of nitrate make ammonia production easier and more cost‐effective.^[^ ^156^ ^]^ For instance, low‐level nuclear waste contains high nitrate concentrations that cannot be treated biologically, making electrochemical nitrate reduction a promising method. However, the presence of hexavalent chromium in such wastewaters can hinder the reduction process. In addition, wastewater from the textile industry and contaminated groundwater often contain elevated nitrate levels, but the concentrations are still relatively low for effective electrochemical reduction. As a result, a preconcentration step is often required before electroreduction. Moreover, neutral pH conditions typically lead to low solution conductivity, which further limits the direct utilization of nitrates. Therefore, in industrial‐scale nitrate reduction systems, it is necessary to integrate deoxygenation units, catalyst protection modules, disinfection systems, and pH adjustment units to meet the practical requirements of industrial applications.^[^ ^157^ ^]^ Future research on NO_3_^−^RR should involve systematic studies on real wastewater conditions and explore the effects of multicomponent electrolytes to improve the practicality and scalability of this technology.

### 4.2 Anions and Cations

In the supporting electrolyte for NO_3_^−^RR, in addition to the presence of main acids, bases and salts, excess alkali metal cations, sulfates, halides, and their derivatives significantly affect the reaction. A classic study is that Katsounaros et al. demonstrated that at a potential of −1.8 V (vs Ag/AgCl), an increase in NaCl concentration led to a significant increase in the nitrate reduction rate.^[^ ^159^ ^]^ The order of effectiveness for alkali metal cations in enhancing nitrate reduction is as follows: Li^+^ < Na^+^ < K^+^ < Cs^+^. This was initially attributed to changes in the electric double layer structure, but further research indicated that this effect is actually due to changes in the ψ₁ potential at the plane where anion reduction occurs.^[^ ^160^, ^161^ ^]^

While this theory explains part of the phenomena, it does not fully account for all observed behaviors. Other researchers have introduced the concept of “cation catalysis” to explain how unreacted cations in the supporting electrolyte can influence anion reduction. They argue that the presence of these cations affects the double layer structure by forming ion pairs, reducing the repulsive force between anions and similarly charged electrodes.^[^ ^162^, ^163^, ^164^ ^]^ Multivalent cations, such as Ca^2+^ and La^3+^, have an even greater impact on nitrate reduction efficiency compared to alkali metals. Manzo‐Robledo et al. used real‐time on‐line differential electrochemical mass spectrometry combined with chronoamperometry to study nitrate reduction in K^+^ and Na^+^‐containing electrolyte solutions. Their study found that the HER was influenced by K^+^ and Na^+^ ions. In a K^+^‐containing solution, the HER occurs moderately, and the product of nitrate reduction is biased toward nitrogen. However, in a Na^+^‐containing solution, the HER kinetics are higher than in K^+^‐solution, which suppresses nitrogen generation, shifting the product distribution toward N_2_O.^[^ ^165^ ^]^

**Figure 16:**
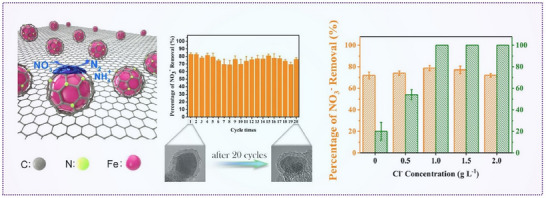
Effect of anions and cations on nitrate reduction performance. Schematic diagram of nitrate reduction by iron nanoparticles encapsulated in N‐doped graphitic carbon (Fe(20%)@N‐C), stability of nitrate reduction and effects of Cl^−^ concentration. Reproduced with permission.^[^ ^158^ ^]^ Copyright 2019, Elsevier.

In addition to cations, the specific adsorption of anions on the catalyst surface can inhibit nitrate adsorption, which, in turn, decreases the reduction rate, and the reduction rate decreases in the order of I^−^ > Br^−^ > Cl^−^ > F^−^ at a potential of −1.8 V.^[^ ^159^ ^]^ Additionally, the presence of Cl^−^ ions can increase nitrogen yield. In a typical nitrate reduction process, nitrate is reduced to ammonia at the cathode, while Cl^−^ ions are oxidized at the anode to form Cl_2_. The Cl_2_ then reacts with water to form ClO^−^, which can reoxidize ammonia to nitrogen. However, high concentrations of Cl^−^ in the solution can reduce nitrate reduction efficiency, likely due to Cl^−^ occupying the catalyst's active sites or the formation of ClO^−^, which reoxidizes nitrate to its original form.^[^ ^36^, ^158^, ^166^, ^167^ ^]^ For example, Duan et al. developed a class of highly active and durable electrocatalysts for nitrate reduction. The unique Fe(20%)@N*─*C electrocatalyst, consisting of iron nanoparticles encapsulated in N‐doped graphitic carbon, achieved a nitrate removal rate of 83.0%. In the absence of Cl^−^, the nitrogen selectivity was 25.0%, which increased to 100% upon the addition of 1.0 g L^−1^ NaCl. Moreover, the catalyst exhibited excellent stability, with no statistically significant difference in the removal percentage recorded over 20 cycles (**Figure** **16**).^[^ ^158^ ^]^ To avoid this undesirable cross‐oxidation, researchers have optimized the electrolytic cell configuration, with dual‐chamber cells proving to be more efficient than single‐chamber cells for nitrate reduction.^[^ ^168^ ^]^

In laboratory conditions, nitrate reduction is often conducted in solutions with relatively simple compositions, which means the yield and conversion rates are less influenced by external factors. However, in real‐world applications, nitrate‐containing solutions are much more complex. The regulation of variables such as nitrate concentration, solution pH, and the presence of anions and cations is crucial. A better understanding of how these parameters affect nitrate reduction will improve our ability to effectively manage nitrate‐containing wastewater in practical applications.

## 5 Protocols for Accurate NO_3_^−^RR Evaluation

### 5.1 Detection of Ammonia in NO_3_^−^RR

Ammonia is often considered the valuable product in NO_3_^−^RR, while other products are viewed as by‐products. As a result, accurately detecting ammonia is crucial. The most commonly used method for ammonia detection is colorimetry, specifically the indophenol blue method, which relies on the Lambert–Beer law. This technique uses the specific absorption wavelength of ammonia in the ultraviolet–visible (UV–vis) spectrum to quantify its concentration. According to recent studies, over 90% of ammonia detection is performed using UV–vis spectrophotometry. Besides ammonia, this method can also be employed to detect other ions like nitrate, nitrite, and hydroxylamine.^[^ ^169^, ^170^, ^171^, ^172^ ^]^

Another effective method for detecting ions in the electrolyte is ion chromatography (IC). Cation chromatography is used to measure the NH_4_^+^ concentration, while anion chromatography is employed for detecting nitrate and nitrite concentrations.^[^ ^78^ ^]^ Similar to UV–vis spectrophotometry, IC requires the preparation of a standard curve using known solutions for accurate measurements. For real‐time ammonia concentration detection, an ammonia meter can be directly employed, offering a more convenient approach.^[^ ^133^ ^]^ For the quantification of volatile products and Faradaic efficiency of volatile gases, gas chromatography‐mass spectrometry (GC‐MS) is used. To exclude external nitrogen contamination and ensure the purity of the ammonia measured, isotope labeling experiments and nuclear magnetic resonance (NMR) are essential. Typically, ^15^NO*_x_* (99 atom%) is used as the nitrogen source,^[^ ^173^ ^]^ and after the reaction, ^1^H‐NMR is used to detect ^15^NH_4_^+^ in solution. This allows for accurate tracing of the ammonia produced from nitrate reduction.

### 5.2 Key Performance Parameters for Catalysts

Several parameters are used to evaluate the performance of catalysts for nitrate reduction, with the ammonia yield rate being a key factor. The ammonia production rate per unit time, area, or mass is critical for assessing the economic viability of the catalyst. The conversion rate of nitrate is also important, especially when considering nitrate pollution removal. It is defined as the ratio of nitrate converted to total nitrate in the original solution.

The selectivity of the catalyst refers to the proportion of ammonia produced in the total nitrogen reduction. The goal of current research is to develop catalysts that exhibit both high selectivity and high ammonia yield. The Faradaic efficiency represents the ratio of charge required to synthesize ammonia to the actual charge consumed by the catalyst during the reaction. A high Faradaic efficiency indicates fewer competing side reactions, such as the hydrogen evolution reaction, which can significantly lower catalyst efficiency.

### 5.3 Evaluation Formulas

(1) $$\mathrm{Yield}\mathrm{rate}:\mathrm{Yield}=\left(c\times V\right)/\left(t\times S\right)\mathrm{or}\left(c\times V\right)/\left(t\times m\right)$$

(2) $$\mathrm{Conversion}\;\mathrm{rate}:\;\mathrm{Conversion}=\Delta c_{\mathrm{NO}3-}/c_{0}\times 100\%$$

(3) $$\mathrm{Selectivity}\;\mathrm{of}\;\mathrm{the}\;\mathrm{product}:\;\mathrm{Selectivity}=c/\Delta c_{\mathrm{NO}3-}\times 100\%$$

(4) $$\begin{array}{cc} & \mathrm{Faradaic}\;\mathrm{efficiency}:\;\mathrm{Faradaic}\;\mathrm{efficiency}\\ & \;=\left[\left(n\times 96\;485\times c\times V\right)/\left(M\times Q\right)\right]\times 100\% \end{array}$$

where *c* is the concentration of ammonia, *V* is the volume of the solution, *t* is the reaction time, *m* is the quality of catalyst, *S* is the area of supported catalyst, Δ*c*_NO3‐_ is the difference in the concentration of nitrate before and after the reaction, *c*_0_ is the initial nitrate concentration in the solution, *n* is the number of electrons transferred, *M* total mass of ammonia produced, and *Q* is the consumed charge.

### 5.4 Evaluation of Catalyst Active Sites

In electrocatalysis, it is common to evaluate catalysts based on the number of active sites. One widely used method is calculating the electrochemically active surface area (ECSA). Some techniques, such as double layer capacitance, hydrogen underpotential deposition, N_2_O titration, CO stripping, and copper underpotential deposition are employed to determine ECSA, providing insights into the true active sites of the catalyst.^[^ ^49^, ^95^, ^174^, ^175^, ^176^ ^]^ The calculation of ESCA can give us a better understanding of the real active sites of the catalyst. For planar supported catalysts, the normalization of the activity of the geometric area may explain the problem, but for rough catalysts, the normalization of ECSA is more convincing.

### 5.5 Reaction Kinetics

The Tafel slope from LSV can be used to assess the reaction kinetics. The rate‐determining step (RDS) of the reaction can be inferred from the Tafel slope value.^[^ ^177^ ^]^ For NO_3_^−^RR, a Tafel slope close to 120 mV dec^−1^ suggests that the RDS is the reduction of nitrate to nitrite. A Tafel slope greater than 120 mV dec^−1^ indicates that the process involves both chemical and electrochemical steps, where the chemical process is the RDS, followed by rapid electrochemical steps.^[^ ^51^ ^]^

### 5.6 Catalyst Stability and Durability

Catalyst stability is critical for its practical application. During the reaction, intermediate species or adsorbed molecules can block active sites, leading to catalyst deactivation. Thus, evaluating the reuse and durability of catalysts is essential. In‐depth understanding of the catalyst's mechanism and its performance degradation is crucial, which is where in situ techniques come into play. These methods provide real‐time insights into the catalyst behavior and help identify potential areas for improvement.

**Figure 17:**
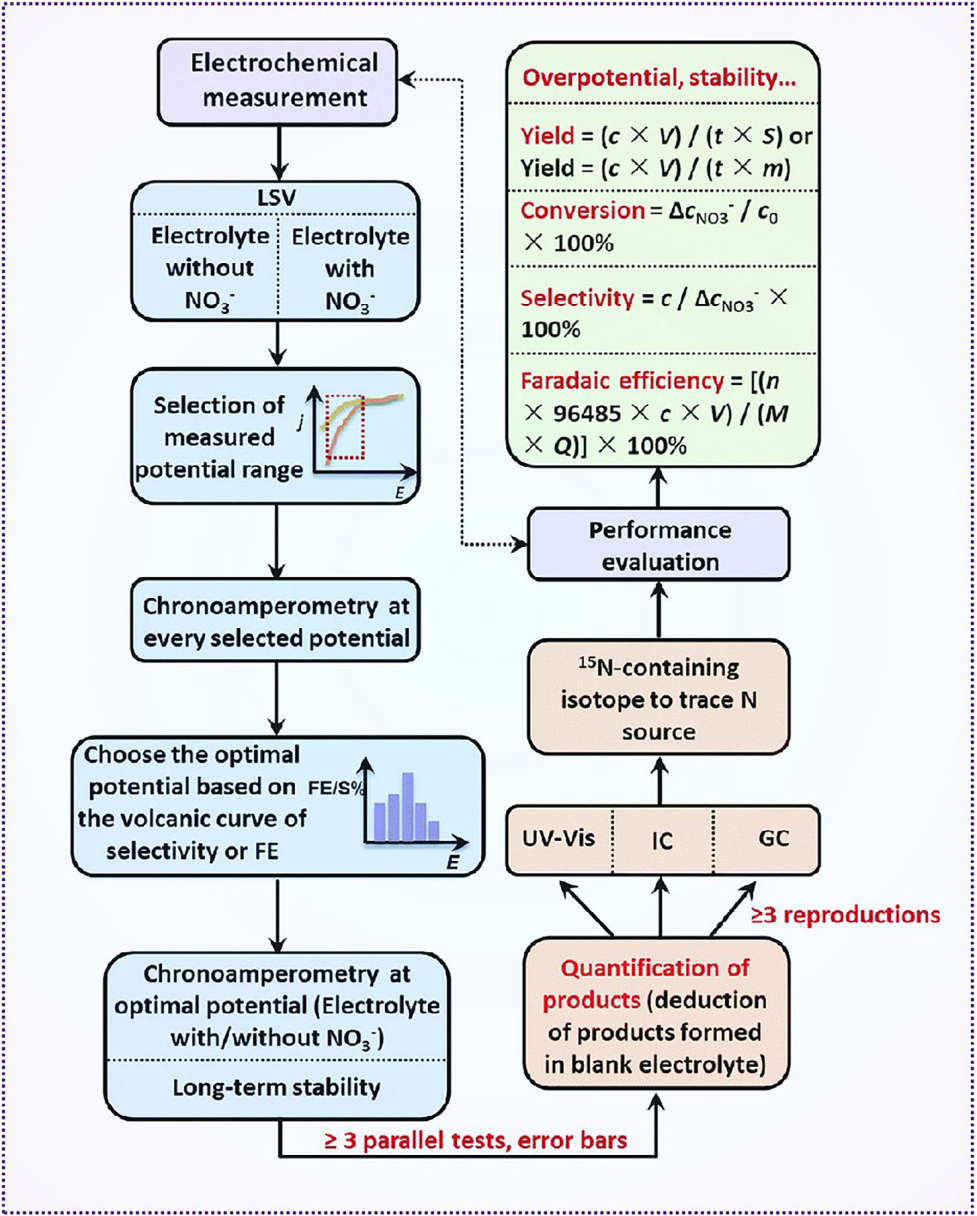
Measurement protocol for assessing NO_3_^−^RR performance. Reproduced with permission.^[^ ^39^ ^]^ Copyright 2021, Royal Society of Chemistry.

Besides, a comprehensive understanding of ammonia production, catalyst evaluation, and reaction mechanisms is essential for improving nitrate reduction catalysis. Current research not only focuses on increasing efficiency but also on understanding the underlying processes through advanced characterization techniques. Wang et al. summarized the current in situ characterization of NO_3_^−^RR and made a standardized recommendation for the test sequence of nitrate reduction, which gave an insightful standardization guidance (**Figure** **17**).^[^ ^39^ ^]^

## 6 Post‐Treatment of Ammonia

During the NO_3_^−^RR process, the generated ammonia is typically present either as ammonium ions (NH_4_⁺) dissolved in the electrolyte or as gaseous NH_3_, depending on the pH and operating conditions. Efficient recovery and quantitative analysis of this ammonia are crucial for evaluating catalytic performance and enabling its subsequent utilization. The most common recovery strategies include distillation, gas stripping, membrane separation, and acid trapping. For example, Huo et al. employed a membrane distillation technique to recover the generated ammonia product, ammonium sulfate, from saline water. In their process, the ammonia‐rich brine was first treated by membrane distillation to remove ammonia from the saline solution. The extracted ammonia was then captured using a H_2_SO_4_ solution and ultimately recovered in the form of (NH_4_)_2_SO_4_, which is a potentially valuable fertilizer product.^[^ ^178^ ^]^ The combination of gas stripping and acid trapping is widely adoptable due to its simplicity, scalability, and cost‐effectiveness. For example, Wei et al. utilized this combined approach to collect and convert ammonia. Taking advantage of the higher vapor pressure of NH_3_ under alkaline conditions, they effectively collected the NH_3_ product through heating and gas stripping. The stripped NH_3_ vapor was then absorbed by HNO_3_ solution and subsequently recovered as NH_4_NO_3_ powder via rotary evaporation. Through this two‐step process, the valuable fertilizer ammonium nitrate was successfully obtained.^[^ ^179^ ^]^

## 7 Challenges and Summary

Considering the multipath, multielectron nature of NO_3_^−^RR, the catalysts, catalytic environment, and catalyst evaluation methods for nitrate reduction are summarize based on current research advances. The enhancement of catalyst performance is discussed from various perspectives, including morphology engineering, crystal facet engineering, strain engineering, defect engineering, alloying engineering, compositional engineering, doping engineering, confinement engineering, and biomimetic engineering. The electrolyte, which is integral to the catalytic environment, significantly influences performance, with key factors, such as pH, nitrate concentration, and the roles of anions and cations affecting catalytic efficiency. Also, the detection of ammonia, the desired product of nitrate reduction, remains a critical aspect of research. We provide an overview of current detection methods and standardize key parameters for catalyst evaluation, including Faradaic efficiency, ammonia yield, selectivity, conversion rate, and stability. **Table** **1** summarizes recent catalyst materials, synthesis methods, electrolyte types, and concentrations, ammonia production rates, Faradaic efficiencies, product types, selectivity, and ammonia detection methods.

**Table 1:** The NO_3_⁻RR performance of various catalysts.

Catalyst	Synthesis method	Electrolyte	NH^4+^ yield [%]	FE [%]	Production	Selectivity [%]	Detection method	Refs.
NiCo_2_O_4_/CC A1	Hydrothermal and annealing	0.1 m NaOH + 0.1 m NaNO3	973.2 µmol h^−1^ cm^−2^ at ‐0.3 V	99.0 at ‐0.6 V	NH_3_, NO_2_^−^		Colorimetric method	[75]
Pt_78_Ru_22_/C	NaBH_4_ reduction	1 m H_2_SO_4_ + 1 m NaNO_3_		>93	NH_3_, NO		Indophenol blue method	[95]
2H Rh NPLs	Solvothermal	1 m KOH + 1 m KNO_3_	156.97 mg h^−1^ mg_cat_^−1^	91.9	NH_3_		Indophenol blue method and ^1^H NMR	[77]
Rh/C	Commercial electrocatalysts	0.1 m KOH + 0.1 m KNO_3_	34.4 µg h h^−1^ cm^−2^ at 0.1 V	20.8	NH_3_		Indophenol blue method	[80]
Ni*─*CuO	Thermal calcination	0.5 m Na_2_SO_4_ +0.1 m KNO_3_	0.94 mmol h^−1^ cm^−2^	95.26	NH_3_	90	Indophenol blue method	[111]
Cu‐Pt foam	Electrodeposition	0.1 m Na_2_SO_4_ +10 mm NaNO_3_	194.4 mg NH_3_‐ N L^−1^gcat^−1^	22	NH_3_	84	Colorimetric method	[101]
Cu_2_O/CP	Liquid deposition	0.5 m Na_2_SO_4_ + 200 ppm NaNO_3_	0.0699 mmol h^−1^ mg^−1^	85.26	NH_3_	85.78	Colorimetric method	[88]
Pd‐Cu_2_O	Water bath	0.5 m K_2_SO_4_ + 50 ppm KNO_3_	925.11 µg h^−1^ mg_cat_^−1^	96.56	NH_3_	95.31	Colorimetric method	[89]
Cu@Cu_2+1_O NWs	Water bath	0.5 m K_2_SO_4_ + 50 mg L^−1^ KNO_3_	576.53 µg h^−1^ mg_cat_^−1^	87.07	NH_3_, NO_2_^−^	76	Colorimetric method	[105]
Cu_2_O/Cu	Pulse electrodeposition and electroreduction	1 m KOH +250 mg L^−1^ NaNO_3_	2.17 mg cm^−2^ h^−1^	84.36	NH_3_, NO_2_^−^	94.4	Indophenol blue method	[106]
Fluorine doped carbon	Pyrolysis	0.05 m H_2_SO_4_ +200 ppm KNO_3_	23.8 mmol h^−1^ mg_cat_^−1^	20	NH_3_		Colorimetric method	[122]
Ni(OH)_2_@Ni	Self‐activation	0.1 m Na_2_SO_4_ +200 mg L^−1^ NaNO_3_		64.4	NH_3_	95.5	Colorimetric method	[109]
g‐C_3_N_4_	Thermal calcination	0.5 m Na_2_SO_4_ +100 ppm NO_3_^−^	0.03262 mmol^−1^ g^−1^ h^−1^	89.96	NH_3_	69.78	Colorimetric method	[93]
CoO*_x_* nanosheets	One‐pot synthesis	0.1 m KOH +0.1 m KNO_3_	82.4 ± 4.8 mg h^−1^ mg_cat_^−1^	93.4 ± 3.8	NH_3_		Cammonia meter	[133]
TiO_2−_ *_x_*	Electrochemical anodization process and calcination	0.5 m Na_2_SO_4_ +50 ppm NO_3_^−1^	0.045 mmol h^−1^ mg^−1^	85.0	NH_3_	87.1	Colorimetric method	[87]
Cu@Th‐BPYDC	Postsynthetic modification	1 m KOH + 100 mm KNO_3_	225.3 µmol h^−1^ cm^−2^	92.5	NH_3_		Colorimetric method	[126]
Fe‐cyano NSs	Ice‐templated method	1 m KOH + 0.1 m KNO_3_	42.1 mg h^−1^ mg_cat_^−1^	90	NH_3_		Indophenol blue method	[68]
CuO nanowire	Anodic oxidation and thermal treatment	0.1 m Na_2_SO_4_ +200 ppm NaNO_3_	0.2449 mmol h^−1^ cm^−2^	95.8	NH_3_	81.2	Colorimetric method	[104]
CuCl_BEF		0.5 m Na_2_SO_4_ +100 mg L^−1^ NO_3_^−1^	1.82 mg h^−1^ cm^−2^	44.7	NH_3_, NO_2_^−^	98.6	Colorimetric method	[134]
Ni_3_B@NiB_2.74_	Wet chemistry	0.10 m KOH + 10 mm NO_3_^−1^	198.3 mmol cm^−2 ^h ^−1^	100	NH_3_		Indophenol blue method	[100]
ZnCo_2_O_4_	Hydrothermal and calcination	0.10 m KOH + 100 mm KNO_3_	2100 µg mg^−1^ h ^−1^	95.4	NH_3_		Indophenol blue method	[76]
Cu_49_Fe_1_	Electrodeposition	0.1 m K_2_SO_4_ + 2 mm KNO_3_	0.23 mmol h^−1^ cm^−2^	94.5	NH_3_	86.8	Colorimetric method	[102]
Fe‐PPy SACs	Calcination	0.1 m KOH + 10 mm KNO_3_	2.75 mg h^−1^ cm^−2^	100	NH_3_, NO_2_^−^	≈100	Indophenol blue method	[127]
Pd/TiO_2_	Seed‐assisted approach and ion exchange	1 m LiCl+0.25 m LiNO_3_	1.12 mg cm^−2^ h^−1^	92.1	NH_3_, NO_2_^−^, N_2_H_4_		Indophenol blue method	[85]
Fe_3_O_4_ /SS	Anneal	0.1 m NaOH + 0.1 m NaNO_3_	10145 µg h^−1^ cm^−2^	91.5	NH_3_, NO_2_^−^		Indophenol blue method	[110]
Cu_50_Ni_50_	Electrodeposition	1 m KOH + 0.1 m KNO_3_		99 ± 1	NH_3_		Indophenol blue method	[94]
Ru‐CuNW	Cation exchange method, anneal strategy and in situ electrochemical prereduction	1 m KOH + 2000 ppm NO_3_^−^	76500 µg h^−1^ cm^−2^	96	NH_3_, NO_2_^−^	99.8	Indophenol blue method	[180]
Fe SAC	TM‐assisted carbonization, Pyrolysis and etching	0.25 m K_2_SO_4_ + 0.5 m KNO_3_/0.1 m K_2_SO_4_	20 000 µg h^−1^ cm_cat._^−2^	≈75	NH_3_, NO_2_^−^, N_2_, H_2_		Indophenol blue method	[124]
O‐Cu*─*PTCDA	Autoreduction	0.1 m PBS +500 ppm NO_3_^−^	436 ± 85 µg h^−1^ cm^−2^	77 ± 3	NH_3_, NO_2_^−^, H_2_		Indophenol blue method	[132]
Bi−Cl_red_	Solvent‐free mechanical grinding and electrochemical reduction	1 m KOH + 0.5 m KNO_3_	46.5 g h^−1^ g_cat_^−1^	90.6	NH_3_, NO_2_^−^		Colorimetry method	[82]
CuCoSP	Electrochemically conversion	1 m KOH + 0.01 m NO_3_^−^	1.17 mmol cm^−2^ h^−1^	93.3 ± 2.1	NH_3_, NO_2_^−^		Indophenol blue method	[129]
RuO_2_ Nanosheets	Molten salt	0.5 m Na_2_SO_4_ + 200 ppm m NaNO_3_	0.1158 mmol h^−1^ cm^−2^	97.46	NH_3_	96.42	Colorimetry method	[67]
Pd‐NDs/Zr‐MOF	In situ reduction	0.1 m Na_2_SO_4_ +500 ppm NO_3_^−^	287.31 mmol h^−1^ g_cat_^−1^	58.1	NH_3_		Indophenol blue method	[73]
GaInSn	Liquid‐metal	0.1 m HNO_3_	2335 µg h^−1^ cm^−2^	100	NH_3_		NMR	[98]
Intermetallic CuPd nanocubes	Colloidal method	1 m KOH + 1 m KNO_3_	6.25 mol h^−1^ g^−1^	92.5	NH_3_, NO_2_^−^		Colorimetric method	[58]
TiO_2_ NTs/CuO*_x_*	Anodization, electrodeposition and calcination	0.5 m Na_2_SO_4_ +100 ppm NO_3_^−^	1241.81 µg h^−1^ cm^−2^	92.23	NH_3_, NO_2_^−^		Indophenol blue method	[107]
Ru&Cu/Cu_2_O	A modified solvothermal	1 m KOH +0.1 m KNO_3_	1 mmol h^−1^ cm^−2^	95	NH_3_, NO_2_^−^	90.4	Colorimetric (indophenol blue) method	[108]
Cu−Pd/C	Heating reduction	0.1 m KOH +10 m NO_3_^−^	220.8 µg mg_cat_^−1^ h^−1^	62.3	NH_3_, NO_2_^−^		Indophenol blue method	[103]
Cu−N−C SAC	Chemical oxidation and modified procedure	0.1 m KOH +0.1 m KNO_3_	4.5 mg cm^−2^ h^−1^ (12.5 mol _NH3_ g_Cu_^−1^ h^−1^)	84.7	NH_3_, NO_2_^−^		Indophenol blue method	[125]
BCDs/NiCo_2_O_4_/carbon cloth	Hydrothermal reaction and subsequent high‐temperature‐annealing	0.5 m K_2_SO_4_ + 200 ppm NO_3_^−^	173.9 µmol h^−1^ cm^−2^	≈100	NH_3_		Ion chromatograph	[116]
Cu nanotubes	Water bath and electroreduction	0.5 m K_2_SO_4_ + 50 mg L^−1^ NO_3_^−^	778.6 µg h^−1^ mg^−1^	85.7	NH_3_, NO_2_^−^	86.2%	Colorimetric method	[72]
CuCo_2_O_4_/CFs	Electrospinning and high temperature carbonization	1.0 m KOH +0.1 m NO_3_^−^	394.5 mmol h^−1^ g^−1^	81.9	NH_3_, NO_2_^−^		Indophenol blue method	[74]
SN Co‐Li^+^/PCNF	Electrospinning and subsequently pyrolysis process	0.5 m Na_2_SO_4_ + 0.5 m NO_3_^−^	2.1 mmol h^−1^ cm^−2^	90.2	NH_3_, NO_2_^−^		Indophenol blue method	[181]

Despite significant advances in nitrate electroreduction, several challenges persist, including high catalyst costs, low Faradaic efficiencies, high overpotentials, competing adsorption reactions, and long‐term catalyst instability. Recent in situ characterizations and theoretical calculations have greatly enhanced our understanding of the catalytic mechanisms, providing valuable insights for the design of catalytic materials and improving the selectivity of catalysts for ammonia production. This progress has moved the field beyond a trial‐and‐error approach, offering a more informed direction for future research.

To accelerate the development of more efficient nitrate‐reducing nanomaterials, we conclude by highlighting the current challenges and future opportunities in the field of nitrate electroreduction.

### 7.1 Catalyst Design

The design of inorganic catalysts for NO_3_^−^RR is primarily guided by theoretically calculated adsorption energies and energy barriers required for the catalytic pathways. Currently, most nitrate reduction catalysts are based on copper‐based materials. However, metal nanomaterials, especially single‐metal nanomaterials, are prone to poisoning during the reaction, limiting their efficiency. Unconventional crystal phase engineering also provides a novel and promising pathway to improve the electrocatalytic performance for NO_3_^−^RR by fundamentally altering the coordination environment, electronic structure, and surface atom arrangements of catalysts. Compared with conventional crystalline phases, metastable or amorphous‐like phases often exhibit enhanced charge transfer capability, more unsaturated surface sites, and flexible geometric structures, which are beneficial for the adsorption and activation of NO_3_⁻ and its intermediates. Overall, leveraging unconventional crystal phases offers a unique pathway to overcome the current limitations in NO_3_⁻ electroreduction. Future efforts should focus on stabilizing metastable phases, modulating the electronic structure of unconventional phases, and elucidating the structure–activity relationships associated with these nontraditional crystal structures. In nature, the active centers of nitrate reductase are predominantly composed of Mo, Co, and Fe, suggesting that biomimetic approaches could be beneficial. By modeling metalloenzymes at the atomic level, it is possible to create catalysts with active sites that promote secondary interactions, enhancing electrocatalytic performance for nitrate reduction. Despite this potential, biomimetic metalloenzymes are still underexplored, and understanding the relationship between enzyme structure and performance is critical. A tandem catalyst for nitrate reduction takes advantage of enzyme‐like functionality to achieve efficient nitrate conversion. This provides valuable insights into the design of composite materials, which could further enhance the selectivity and efficiency of nitrate reduction processes.

### 7.2 Unified Evaluation Criteria

While there is a strong call within the electrocatalysis community for standardized testing and evaluation criteria, many existing studies fail to provide intuitive, comparable assessments of catalyst quality. As highlighted in Table 1, recent literature shows a lack of standardization regarding the units for ammonia production rates, as well as an absence of optimized procedures for selecting electrolyte pH and nitrate concentration. Without uniform testing conditions, the performance of different catalysts cannot be reliably compared, and high ammonia production rates alone are insufficient for evaluating catalyst efficacy. Factors such as electrolyte pH control and potential selection must also be considered, especially with respect to their impact on energy consumption.

### 7.3 Practical Application

Most studies on NO_3_^−^RR are conducted using synthetic solutions, neglecting the complex interactions that occur in real‐world applications with natural water components. As discussed, the anions and cations in the electrolyte can significantly influence the catalytic reaction. From a practical standpoint, it is essential to evaluate nanocatalysts using real nitrate‐containing wastewater samples, as this would provide more accurate insights into long‐term catalyst stability and ammonia collection efficiency. Furthermore, practical evaluations should also account for the economic value of the process, such as the potential to recover valuable by‐products. Electrolytes containing elements like nitrogen (N), phosphorus (P), and potassium (K) could also be recycled as fertilizers, adding value to the process.

### 7.4 Anodic Coupling Reactions

With the trend toward multifunctional devices, it is possible to reduce energy consumption and generate valuable by‐products by coupling the NO_3_^−^RR with other anodic reactions. While NO_3_^−^RR is typically a cathodic process, the corresponding oxidation of molecules at the anode can also be leveraged, depending on the catalyst material used. This concept is similar to electrolytic water splitting, where anodic oxidation reduces energy consumption. For instance, Niu et al. coupled nitrate reduction with methanol oxidation to achieve energy reduction,^[^ ^74^ ^]^ demonstrating the potential for integrated systems that utilize modified or engineered nanocatalysts to operate efficiently across a broader range of platforms.

### 7.5 Mechanism Exploration

The NO_3_^−^RR in aqueous solutions involves a complex proton and electron pair transport pathway, which is distinct from processes like the Haber–Bosch synthesis of ammonia. The rate‐limiting steps, as well as the selective adsorption of nitrate, significantly influence catalyst design, selectivity, and energy consumption. However, the detailed reaction mechanism remains difficult to elucidate due to the involvement of numerous unstable intermediates and products. To gain a deeper understanding of the catalytic process, advanced in situ characterization methods, including differential electrochemical mass spectrometry (DEMS), Fourier transform infrared spectroscopy (FTIR), ion chromatography (IC), electron spin resonance (ESR), shell‐isolated nanoparticle‐enhanced Raman spectroscopy (SHINERS), scanning tunneling microscopy (EC‐STM), and X‐ray absorption spectroscopy (XAS), are essential. These techniques, either individually or in combination, can provide valuable insights into reactive intermediates and species during nitrate reduction. Additionally, DFT calculations are instrumental in simulating interactions between reaction intermediates and active species, offering predictive power for catalyst design and optimization.

### 7.6 Advanced Electrochemical Device Construction

Despite recent progress in the development of NO_3_^−^RR, the construction of advanced electrochemical devices still faces multiple technical challenges. One key obstacle lies in designing reactor systems that can efficiently accommodate gas–liquid–solid interfaces while ensuring high mass transfer rates and stable operation. Traditional H‐type cells suffer from high internal resistance and limitations in scalability and practicality, thus more advanced systems, such as flow cells or membrane electrode assemblies (MEAs), are required. These devices should integrate optimized electrode architectures, precise electrolyte management, and real‐time product separation modules without compromising performance or increasing system complexity. Due to the high cost of membrane materials, membrane‐free reactors have also emerged as a promising direction for device development. Additionally, integrated systems such as the nitrate‐zinc battery have demonstrated multifunctionality by enabling electricity generation, ammonia production, and wastewater treatment simultaneously. This multifunctionality highlights the potential of incorporating NO_3_^−^RR into broader energy device platforms.

## 8 Conclusion and Outlook

As an essential part of the nitrogen cycle, NO_3_^−^RR faces both significant opportunities and challenges moving forward. A deep understanding of the nitrate reduction process at the molecular level is crucial for advancing ammonia production technologies, which holds great promise for both scientific research and industrial applications. Despite existing challenges, the development of efficient, low‐cost, and stable catalysts is pivotal for addressing both environmental and energy concerns. This review provides a comprehensive overview of catalytic materials, engineering approaches, evaluation systems, and influential factors, aiming to offer valuable guidance for future developments in nitrate reduction catalysis.

## Conflict of Interest

The authors declare no conflict of interest.
